# Microbial allies in skin trauma recovery: from immune modulation to engineered probiotic therapeutics

**DOI:** 10.1093/burnst/tkaf068

**Published:** 2025-10-23

**Authors:** Aline Yen Ling Wang, Ana Elena Aviña, Yen-Yu Liu, Huang-Kai Kao

**Affiliations:** Center for Vascularized Composite Allotransplantation, Chang Gung Memorial Hospital, No. 5, Fuxing St., Guishan District, Taoyuan City 333, Taiwan, China; Center for Vascularized Composite Allotransplantation, Chang Gung Memorial Hospital, No. 5, Fuxing St., Guishan District, Taoyuan City 333, Taiwan, China; International PhD Program in Medicine, College of Medicine, Taipei Medical University, 250 Wuxing Street, Taipei City 110, Taiwan, China; Center for Vascularized Composite Allotransplantation, Chang Gung Memorial Hospital, No. 5, Fuxing St., Guishan District, Taoyuan City 333, Taiwan, China; Department of Plastic and Reconstructive Surgery, Chang Gung Memorial Hospital, No. 5, Fuxing St., Guishan District, Taoyuan City 333, Taiwan, China; College of Medicine, Chang Gung University, No. 259, Wenhua 1st Rd., Guishan District, Taoyuan City 333, Taiwan, China

**Keywords:** Microbiome, Probiotics, Engineered probiotics, Immunoregulation, Skin wound healing, Trauma recovery

## Abstract

Research shows that the microbiome of the skin is present as an active contributor to wound healing processes by moving past its historical infection-related function. The review investigates how commensal and probiotic bacteria affect immunomodulation while accelerating epithelial growth, together with tissue repair processes. Researchers use modern methods to link immunological concepts with material science along with synthetic biological techniques to study engineered probiotics which transform current wound treatments. The research study represents an extensive integration of recent findings concerning probiotic-mediated immunomodulatory operations and engineered approaches that improve probiotic delivery systems and their performance during skin wound healing procedures. Recent genetically engineered *Lactobacillus reuteri* strains that express chemokines like CXCL12 have been found to promote wound healing to an accelerated rate in animal models, and pre-clinical phases of clinical trials in the setting of diabetic foot ulcers (DFU) has demonstrated safety and therapeutic potential. Simultaneously, another live biotherapeutic product has been validated in terms of regenerative and immunomodulatory properties in animal models and in a clinical trial, a multi-cytokine-integrated strain of *Lactococcus cremoris* secreting FGF-2, IL-4, and CSF-1 promoted faster wound healing in diabetic mice and healed 83% of subjects in a Phase I DFU study. The range of probiotic therapies for trauma care expands due to advancements in probiotic delivery using materials and membrane vesicles derived from probiotics. This review builds a detailed framework that connects core immune functions with modern engineering methods for developing smart wound healing systems that combine engineered probiotics with bioresponsive materials and real-time monitoring systems. Engineered probiotics promise to become an alternative strategy for treating chronic wounds and infection-related complications that currently create significant medical problems.

## Graphical Abstract

**Graphical Abstract:**
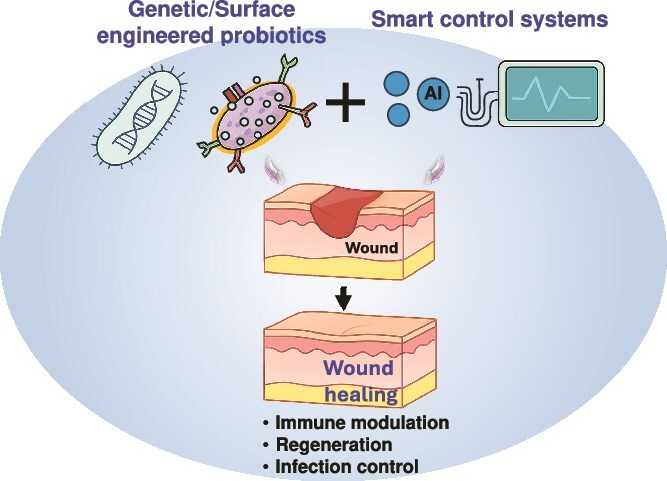

## Highlights

- Engineered probiotics are emerging as active agents in wound healing, capable of modulating immune responses, enhancing epithelialization, and combating infections.
- This review comprehensively integrates key advances in probiotic-mediated immunomodulatory mechanisms and engineering strategies designed to enhance probiotic delivery and function in skin wound healing.
- A CXCL12-producing engineered *Lactobacillus reuteri* strain demonstrated accelerated wound repair in murine, minipig, and human clinical studies, and is now progressing through Phase II trials.
- A multi-cytokine-engineered *Leuconostoc cremoris* secreting FGF-2, IL-4, and CSF-1 accelerated wound healing in diabetic mice and achieved 83% complete closure in a Phase I DFU trial, confirming its regenerative and immunomodulatory potential.
- Smart wound healing platforms integrating engineered probiotics, responsive biomaterials, and real-time sensing technologies represent a transformative frontier in trauma care.

## Background

Current clinical medicine faces an ongoing challenge to achieve effective recovery for all types of skin trauma procedures, including acute injuries and surgical wounds, as well as persistent ulcers. Currently, >10% of diabetic patients develop chronic wounds, and 80% of these wounds recur, leading to a high risk of amputation and substantial healthcare burden [1]. Diabetic foot ulcers (DFU), together with other chronic wounds, present a medical challenge globally because their mortality rate reaches cancer-levels [2]. The rising global traumatic injuries and increasing chronic non-healing wounds among older people with multiple health conditions require immediate innovation of alternative therapeutic methods. Modern wound care technologies have not eliminated the widespread challenges faced by persistent wounds which end in related complications such as infections and scarring or ultimately limb loss. Researchers have responded to unmet clinical requirements by reevaluating wound healing biological processes and the neglected microbiome they find in the affected ecosystem [3, 4].

Medical discourse has normally examined the skin microbiota through a disease-centric viewpoint by treating wound-colonizing bacteria as healing obstacles that serve as targets for severe antimicrobial methods [5–12]. Recent scientific discoveries depict the skin microbiota from a different standpoint. Scientists have discovered that the immune response mechanisms and tissue regeneration abilities of commensal (commensals are native, non-pathogenic microbes residing on the skin) and probiotic bacteria (probiotics are externally introduced beneficial strains with therapeutic potential) continue to grow in evidence [2]. Recognition of host-microbiota interactions has been developed as a major factor of immune homeostasis and tissue resilience. Belkaid *et al.* discussed how commensal microbes coordinate epithelial integrity, innate immune tuning, and development of regulatory T cells (Tregs), which are the key to immune homeostasis in non-pathogenic settings in 2017 [13]. When skin bacteria like *Staphylococcus epidermidis* (*S. epidermidis*) reside on the skin surface, they produce amine metabolites which help keratinocytes adapt to adrenaline stress hormones and support skin barrier maintenance while accelerating wound healing [14]. Wound healing therapeutic potentials exist in common skin commensals *S. epidermidis*, along with *Staphylococcus hominis*, *Staphylococcus lugdunensis*, *Staphylococcus capitis*, *Corynebacterium striatum*, and *Roseomonas mucosa* because they demonstrate immunomodulatory and anti-inflammatory properties [1]. *S. epidermidis* triggers the activation of γδ T cells found in the skin, which then induces perforin-2 (P-2) expression for *Staphylococcus aureus* defense and enhances healing processes through antibacterial peptide secretion and inhibition of pro-inflammatory signals, including interleukin-1β (IL-1β) [3, 4, 15]. The previously threatening microbial residents have now been discovered to partner up with our bodies in their healing operations. This paradigm shift—recognizing the ‘light side’ of the microbiome [3].

Modern research shows how selected bacteria that live on human skin produce immunomodulatory effects and enhance keratinocyte growth and trigger blood vessel formation, which combined create successful wound remediation [1, 16–20]. Studies show that bacteria demonstrate therapeutic potential by using short-chain fatty acids (SCFAs) as mediators and by activating TLR signaling and other immune responses, thus becoming beneficial partners instead of inert observers [21–23]. The deliberate creation and engineering of probiotics to execute therapeutic functions opens a better future as scientists gradually reveal how microbial communities function naturally. Engineered probiotic (probiotics are genetically modified via synthetic biology) therapeutics developed using synthetic biology now enable precise performance of dual detection capabilities along with targeted secretion of growth factors or anti-inflammatory molecules, as well as biomaterial interfacing for delivery purposes. Scientific studies conducted both preclinical and early clinical studies show engineered bacteria achieving effective therapeutic delivery and controlled drug deployment for cancer treatment [24–30]. However, the applications of engineered bacteria in wound healing and trauma treatment remain largely underexplored in this field. An extensive literature evaluation suggested that systematic reviews about engineered bacteria applications for wound healing practice are notably absent from available research. Several existing reviews focus on oncology together with gastrointestinal (GI) disorders and biosensing applications, but current research fails to explore how engineered bacteria technologies can be applied in tissue repair and regeneration [29, 31–36]. Although substance reviews demonstrate the role of engineered bacteria in cancer, GI disorders, and biosensing uses, a study in cancer constitutes an outstanding case of the therapeutic ability of engineered bacteria. Din *et al.* created a synthetic gene circuit to allow *Escherichia coli* populations to undertake synchronized population-dependent lysis cycles in 2016 [37]. Based on synthetic biology technologies, this circuit contained an autoinducer-based loop of positive feedback on the luxI promoter which produces the quorum-sensing molecule acyl-homoserine lactone (AHL). As soon as AHL accumulates to a specific concentration, it interacts with the transcriptional regulator LuxR to create a LuxR-AHL complex that induces the synthesis of lysis gene ϕ174 E, the reporter for fluorescence reporting enzyme sfGFP, and the therapeutic cargo, hlyE (a pore-forming cytotoxin). Such constructed bacteria showed a substantial tumor abatement in murine models by inducing self-destruction and pulsatile drug release when the quorum is reached. Their behavioral programmability is a living delivery platform that can provide a spatiotemporal control of therapeutic deployment. Although designed as a cancer product, this approach has conceptual significance to wound care, in which cyclic, localized delivery of regenerative or antimicrobial molecules can play a major role in wound healing in a protracted situation. The amalgamation of immunobiological and microbiological knowledge with synthetic biological principles leads to a thorough evaluation of natural and engineered probiotic impact on wound healing processes. The research first explores microbial immune regulatory mechanisms before analyzing current and novel probiotic uses in hydrogels and drug delivery, and ends with predictions about engineered probiotics that would suit wound areas specifically.

The review builds its content by moving systematically from essential biological principles to sophisticated uses. Our analysis starts with studying how microbiome interacts with the immunological systems of the host during wound repair before examining microbial effects on healing paths. The study then shifts to review functional clinical uses of natural probiotics for skin wound healing. Unmodified probiotics demonstrate their therapeutic potential through existing practical applications, which create an essential foundation for the main focus of genetically manipulated probiotics that deliver cytokines while promoting angiogenesis and showing self-destruction capabilities upon environmental changes [24–27, 29, 38–42]. A phase I trial using topically administered CXCL12-expressing *Lactobacillus reuteri* (*L. reuteri*) (ILP100) proved engineered probiotics are safe and effective for human wound healing acceleration according to recent clinical data [43]. Another engineered *Lactococcus cremoris* (*Leuconostoc cremoris*) strain (AUP1602-C) that introduced three therapeutic genes, was shown to have a dose–response effect and a good safety profile in a Phase I trial treating non-healing DFU, with complete wound closure at the highest dose observed in 83% of patients [44]. The promising initial findings clash with ongoing obstacles regarding safety issues, regulatory concerns, delivery barriers, and patient receptiveness, which need to be overcome.

This review accomplishes more than state-of-the-art clarification because it fuses interdisciplinary knowledge to showcase upcoming directions while showing potential interconnections. The combination of engineered probiotics with biomaterial scaffolds, which includes intelligent hydrogels, has the potential to develop ‘smart wound healing systems’ that adapt to dynamic changes within the wound environment. The future design of drug delivery platforms and immune modulation systems with microbial sensing capabilities inside unified therapeutic platforms presents an opportunity to revolutionize healing processes in acute and persistent tissue injuries. This review serves a dual purpose to provide information while simultaneously generating inspiration. This review reveals probiotic therapeutic capabilities for trauma healing and it establishes engineered probiotics as emerging role within regenerative medicine processes.

**Table 1:** Comparative analysis of engineered probiotics and biotherapeutic strategies for wound healing

Feature/approach	Engineered probiotics	Phage therapy	Microbiome transplantation	AMPs	Stem cell therapy
Immune modulation	Strong (Treg, M2)	Minimal	Moderate	Limited	Strong (paracrine)
Antimicrobial action	Yes (engineered)	High	Indirect	Direct	Indirect
Delivery	Topical/Oral/Hydrogel	Topical	Topical/Skin graft	Needs carrier	Injection/Topical scaffold
Precision	High (gene circuits)	High	Low	Moderate	Moderate (secretome)
Safety concerns	Genetic transfer risk	Host immune response	Pathogen transfer	Cytotoxicity	Immune rejection, cost

*M2* anti-inflammatory M2 macrophages

**Figure 1:**
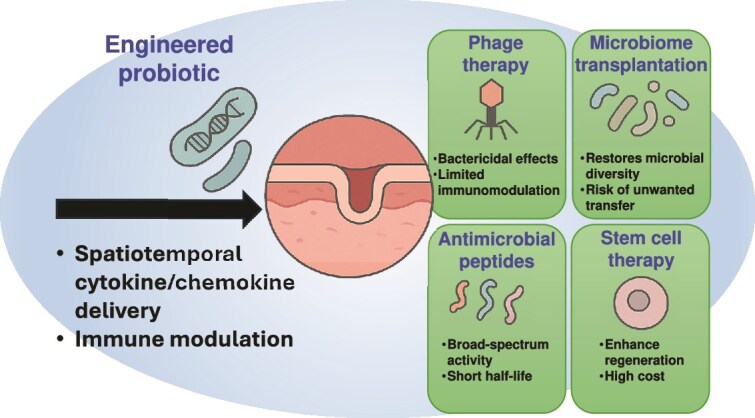
Comparative overview of engineered probiotics and emerging biotherapies for wound care. Probiotics can be engineered to enhance the delivery of cytokines/chemokines/growth factors and immune modulation locally and at short periods of time at wound sites. Compared with phage therapy, microbiome transplantation, AMPs and stem cell therapy, they provide programmable responsiveness and immunomodulatory potential, but there are safety and translation issues to address

### Comparison of engineered probiotics and alternative therapies

In a quest to understand the therapeutic relevance of engineered probiotics better, it is worth taking a look at them within the larger context of emerging wound care alternatives. Conventional therapies have historically been limited in healing chronic wounds but a new generation of biotherapies, including phage therapy, microbiome transplantation, antimicrobial peptides (AMPs), and stem cell-based therapies, may present growing potential. Nevertheless, every one of these approaches has its own tradeoffs with respect to the depth of immunomodulation, abilities to make delivery, specificity, and options to adapt in the clinic. To further manifest these differences, Table 1 provides details of these comparative characteristics among engineered probiotics and phage therapy, microbiome transplantation, AMPs, and stem cell-based therapies. Figure 1 depicts a graphical comparison of benefits and limitations of engineered probiotics and other biotherapies under research in wound healing. An overview of the emerging strategies, though comparative in nature, suggests the versatility of current biotherapeutic applications as well as throws light on the unique opportunity to design probiotics that may unlock therapeutic potential. (i) Engineered probiotics: engineering probiotics act as therapeutic factories that can express cytokines, growth factors, or antimicrobials in a spatiotemporally regulated manner [43–48]. They exhibit stable colonization, self-replication and respond to environmental stimuli (e.g. inflammation, pH, hypoxia) by the responsive secretion of bioactive molecules. The engineered probiotics also help in the homeostasis of immunity systems and the restoration of the epithelial barriers compared to the static ones of drugs. Regulatory issues and biosafety and horizontal gene transfer concerns, however, still persist. There are the benefits of programmable as dual therapeutic and sensing use, and induce immune rebalancing through M2 macrophage polarization, Tregs activation, and can be delivered topically, orally, and via hydrogel. Pluses and minuses surround these techniques as they have potential off-target gene transfer or undesirable ecological perturbations and strain survival and colonization are host- and wound-specific, and clinical translation remains in early phase trials. (ii) Phage therapy: phage therapy is the use of bacteriophages to treat infection, especially antibiotic-resistant infection [49–51]. Phage therapy, though a very exact and potent therapy against biofilms, does not have immunoregulatory effects, and also, it can induce primary immune responses that include neutralizing antibodies [52]. As supported by a recent study, it is possible to show that the neutralizing immune response (including phage-specific neutralizing immunity, especially toward Myoviridae phages) can diminish the therapeutic potential of the Vancomycin-Resistant Enterococcus bacteriophage cocktail in a murine model [52]. In addition, phage resistance and regulatory challenges make it hard to use on chronic wounds. The benefits are that the phages are highly specific to target pathogens such as *S. aureus* and *P. aeruginosa*, and they are active against antibiotic-resistant biofilms [53]. Limitations are that it does not directly have a regenerative or anti-inflammatory effect, and there is poor shelf life and possibility of host immune clearance, and there has to be accurate identification of the pathogen and matching with the phage. (iii) Microbiome transplantation: fecal or skin microbiome transplantation tries to regenerate the well-behaved microbial diversity in chronic or infected wounds [54–58]. Recently, it was shown that fecal microbiota transplantation (FMT) could stage wound healing in diabetic mice to a large extent by altering the composition of the intestinal microbiome and activating the IL-17A-mTOR-HIF-1α signaling pathway, which promoted the migration of keratinocytes, the process of re-epithelialization, and the deposition of collagen [59]. Although this application increases the resilience of microbial communities, it cannot be applied with precision and has the risk of uncontrolled transfer of undesired microbes, particularly in immunocompromised human populations. Contrastingly, transplanted microbiota does not have the goal of selective cytokine production like engineered probiotics. Benefits are increased microbial diversity and suppression of dysbiosis and could restore beneficial species that have immune-regulatory effects. The drawbacks are characterized by donor-to-recipient variations and unpredictable and spontaneous colonization, and the absence of engineered therapeutic attributes. (iv) AMPs: AMPs are small positively charged peptides that demonstrate rather direct bactericidal activity and partially modulate immunity [60–63]. A large number of them are able to improve wound re-epithelialization and decrease the biofilm burden [64, 65]. They are however subject to rapid degradation *in vivo* and high doses are associated with cytotoxicity [66, 67]. In contrast to engineered probiotics, AMPs do not have a long delivery and biological plasticity. The merits are its broad-spectrum antimicrobial action and certain AMPs (e.g. LL-37) stimulate angiogenesis and cell migration [68, 69]. The disadvantages are quick degradation by enzymes in the wound microenvironment, short half-life, and possible cytotoxicity, and demonstrate the need of elaborate delivery vehicles such as hydrogels or liposomes. (v) Stem cell-based therapies: in stem cell-based treatment options, mesenchymal stem cells (MSCs) and other progenitor cells stimulate wound healing through mainly paracrine signaling, angiogenesis and immunomodulation [70–76]. They have proved to be successful in the treatment of chronic diabetic wounds. Stem cell therapy is, however, costly, faces rejection risk from the immune system, and needs special handling. Engineered probiotics provide a non-cellular, although living system, which can provide some paracrine functions. Benefits possess secreted modulatory factors such as vascular endothelial growth factor (VEGF) and IL-10, and decrease inflammation and promote tissue regeneration and angiogenesis. Disadvantages are high price and technical requirements and risk of immune rejection and aging and short shelf-life, and regulatory difficulty.

While each of these alternative therapies (phage therapy, microbiome transplantation, AMPs, and stem cell-based approaches) has its own individual benefits, the limitations also have potential synergies when combined with engineered probiotics. For example, AMPs and phages possess very good antimicrobial activity, but lack immune modulation, which is a deficit that the engineered probiotics may be able to fill by favoring M2 macrophage polarization or Treg activation. Similarly, stem cell therapies have regenerative paracrine effects, which could be complemented by engineered probiotics which locally release growth factors or cytokines in response to wound microenvironmental cues [43, 44]. Microbiome transplantation and engineered probiotics are both aimed at microbial dysbiosis, although the latter is programmable and targeted and avoids variability and safety issues of whole community transfer. Engineered probiotics also provide a more scalable, stable and cost-effective platform of delivery in comparison to the technical and regulatory complexities of stem cell-based therapies. These possible combination strategies would suggest that engineered probiotics do not have to compete with but may potentially augment the effectiveness of other wound healing modalities. Particularly in chronic, multifactorial wounds, such as those in the foot, with ulcers of the diabetic foot or pressure ulcers, a multi-modal approach, which incorporates antimicrobial, regenerative, and immunomodulatory tools, may be most comprehensive and most beneficial. Therefore, instead of being considered as discrete alternatives, engineered probiotics may be better positioned as a central node in a wider therapeutic network, with the capabilities of linking microbial, immunological and regenerative gaps in wound healing. Future research should focus on co-delivery systems, such as hydrogel-based delivery systems or smart scaffolds, incorporating engineered probiotics and AMPs or stem cells to make better use of their synergistic potential [26, 77].

## Review

### Immunomodulatory role of microbiome in trauma recovery

The biological process of wound healing requires a proper balance among all phases, including inflammation together with tissue proliferation, followed by remodeling. This progressive process relies on immune system cells through their continuous interaction between innate and adaptive immune cells to manage pathogen elimination alongside inflammation control and tissue repair stimulation. In the early part of inflammation neutrophils quickly rush to the site of injury, guided by integrins and leukotriene B4 (LTB4) to kickstart the immune response [78]. This has been shown in single-cell analysis to also regulate the organization of the extracellular matrix, by means of the HSF-integrin AM/B2-Kindlin-3 pathway [79]. Interestingly, these neutrophils show diverse gene patterns such as RETNLG, SLC2A3, etc. with some proliferating ones showing BCL2A1B thereby revealing their diverse roles in wound healing [80]. Among the innate immune cells, macrophages hold a central position because they shift their phenotype from early pro-inflammatory M1 to late pro-reparative M2 [81]. M1 macrophages take dominance at the beginning of inflammation because they receive activation signals from danger-associated molecular patterns (DAMPs) molecules in combination with interferon-gamma (IFN-γ) and tumor necrosis factor-alpha (TNF-α) [81]. The M1 polarization process gets activated through transcription factors NF-κB and STAT1 [82–84]. Macrophages in this state generate substantial amounts of pro-inflammatory cytokines IL-1β, IL-6, and TNF-α while performing pathogen and cell death phagocytosis. The M1 macrophages support their antimicrobial functions and inflammatory responses through the Warburg effect, which triggers aerobic glycolysis while simultaneously producing rapid ATP along with reactive oxygen species (ROS) and nitric oxide (NO) molecules [85]. Environmental indicators, including interleukin-4 (IL-4), interleukin-10 (IL-10), and transforming growth factor-beta (TGF-β), together with efferocytosis of apoptotic neutrophils, reshape macrophages into M2 cells [81, 86]. Reported studies validate that transcription factor mechanisms involving signal transducer and activator of transcription 6 (STAT6) and peroxisome proliferator-activated receptor gamma (PPAR-γ) regulate M1 to M2 phenotype transition due to their activation [82–84]. In addition, single-cell RNA sequencing has provided us with a clearer picture of the regulation of wound macrophages. Wu *et al.* found distinct types of macrophages in skin wounds, with their functions determined by important transcription factors such as IRF5, MAFB, and STAT1, which are associated with inflammation, tissue repair, and antigen presentation [87, 88]. Another single-cell study also revealed a macrophage subgroup characterized by the activity of NR4A1 and NFKB1, which likely represents the early responder subset that drives inflammation and attracts neutrophils in early stages of healing. M2 macrophages use oxidative phosphorylation and fatty acid oxidation to generate their energy primarily [85]. The anti-inflammatory cytokines IL-10 and the growth factors VEGF and TGF-β, and platelet-derived growth factor (PDGF) that M2 macrophages produce encourage fibroblast activation, tissue regeneration, along with angiogenesis and extracellular matrix remodeling [81, 86]. In diabetic or chronic wounds, this M1-to-M2 transition is impaired [89–91]. Hyperglycemia combined with advanced glycation end-products creates metabolic dysregulation of macrophages along with decreased efferocytosis function which sustains NF-κB signaling, thus leading to persistent M1 polarization and prolonged inflammation and elevated TNF-α levels while delaying wound healing [89–91]. More recently, single-cell RNA sequencing recently revealed that late-stage macrophages in mice uptake the Wnt inhibitor SFRP4 that maintains chronic Wnt signaling and facilitates fibrotic healing. This shows us an important mechanism of how there is continuing inflammation and tissue scarring of wounds that do not heal [92]. In addition, new data on single-cell RNA sequencing of keloid tissues has characterized a Schwann-like population of fibroblasts has associated with fibrotic wound risks [93, 94]. These cells also interact with the harmful macrophage that releases inflammatory signals and produces the TGF-β signaling mechanism, suggesting a relationship between the activity of nerve stromal cells and immune imbalance in chronic or abnormal wound healing. Wound resolution with proper scar modulation becomes possible through proper regulation of macrophage metabolism

**Figure 2:**
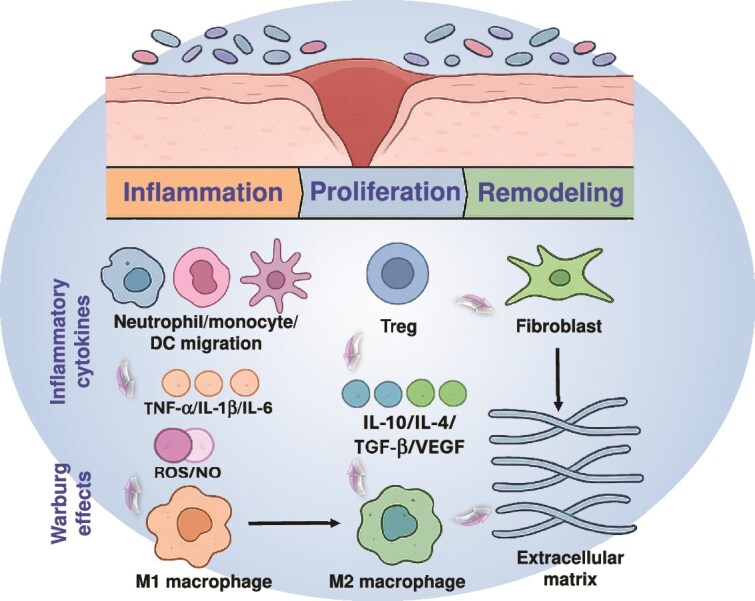
Immune cell transitions during wound healing in the presence of skin microbiota. The figure presents major cellular actors and signaling molecules in the processes of wound healing phases of inflammation, proliferation, and remodeling. Skin microbiota suggests a larger modulating environment that regulates cytokine secretion, macrophage polarization, and tissue repair dynamics

and timely phenotypic changes of these cells while managing vital signaling pathways [81, 86]. Figure 2 gives a conceptual overview of the dynamics of immune cells in the 3 canonical phases of wound healing, the inflammatory, proliferative, and remodeling phases, with integration of the role of the skin microbiome as a modulating factor. The figure shows crucial immune transitions, including recruitment of neutrophils and monocytes during inflammation, M1 to M2 macrophage polarization as well as the subsequent activation of fibroblasts and extracellular matrix deposition during tissue remodeling. The presence of cytokines such as TNF-α, IL-6, and IL-1β in the first phase is shown to lead to inflammatory cascades, and the presence of anti-inflammatory mediators (e.g. IL-10, IL-4, TGF-β) promotes a progression to resolution and tissue repair. Notably, the Figure includes Tregs and the Warburg effect of M1 macrophage and reveals the intersection of immunometabolism and wound healing—an emerging area of therapeutic interest. The integration of skin microbiome as a contextual backdrop in this Figure highlights its dynamic role of causing varying influences on the function of immune cells, cytokines, and potentially tips the balance of issues surrounding chronic inflammation or resolution. This visualization underscores the fact that microbiome-host immune interactions are not on the periphery but are central to the determination of the trajectory of a wound. In mapping the complex immunological pathways in a phase-specific way, the figure will help researchers and clinicians to identify key intervention points that could be targeted to help improve healing outcomes, such as encouraging M2 macrophage, Treg polarization, or microbiome composition. The theoretical framework also lends itself to the development of precision biotherapeutics, such as those engineered probiotics, that can be timed or targeted to specific immunological states within the wound microenvironment. Moreover, single-cell RNA sequencing has also grown our comprehension of how monocytes are converted into macrophages in the process of wound healing. For example, Wee *et al.* identified an important Angptl4-ifi202b signaling pathway driving this transition, and that disruptions of this resulted in lingering neutrophils and delayed healing [95]. Similarly, by using single-cell analysis, Pang *et al.* similarly uncovered a growing population of Ly6C^+^F4/80^low/−^ monocytes/macrophages in diabetic wounds, induced by high CCL2/CCR2 signaling [96]. This impaired the healing process and caused prolonged inflammation with CCL7 further influencing the recruitment and transition of these cells [97]. Using single cell and bulk transcriptomics, researchers determined TFAP2A to be instrumental in the polarization of M2 macrophages that aids in wound healing and new blood vessel formation [98]. The TFAP2A increases LIFR, activating the Hippo-YAP pathway, which pushes macrophages toward a more reparative state with less inflammation in diabetic wounds. Targeting this TFAP2A-LIFR-YAP axis may be helpful to treat chronic inflammation and to enhance healing in DFU.

When interacting with immune cells, the skin microbiota also directly develops adaptive immune responses between skin dendritic cells (DCs) and T cells, along with activating innate defense systems [2, 3, 99–101]. The CD103^+^ and CD11b^+^ skin DCs play a central role in how T cells respond to *S. epidermidis* commensal bacteria [3]. The skin DCs trigger the activation of IL-17A-producing CD8^+^ T cells, also known as Tc17 cells, rather than causing inflammation. Skin DCs, along with their essential barrier-strengthening role also protect the body from multiple pathogens via their cross-protection capability [2, 102]. These commensal-specific CD8^+^ T cells are long-lived tissue-resident memory cells capable of producing IL-17A and IFN-γ, and under inflammatory stimuli such as IL-18 or IL-33, they can even produce type 2 cytokines like IL-13. Through their ability to change their phenotype, T cells demonstrate dual-cytokine potential that allows them to shift from inflammatory defense to tissue-regeneration functions after injuries occur. In commensal-dependent models, IL-13 released from Tc17 cells promotes tissue healing, which strengthens its model-specific function for tissue repair. Single cell chromatin studies found human Tregs to be epigenetically prepared for tissue repair, with open regions in the vicinity of genes such as amphiregulin and IL1RL1 [103]. Once activated by skin injury, these Tregs help to restore the epithelium and keep the immune system in balance during wound healing. Active presence of microorganisms enables DCs to activate specific T cells through rapid response to release proper cytokines for tissue repair and inflammation control [2]. The immune circuit transforms commensal microbes into active regulators who determine skin healing potential through their interactions with the inflammatory state.

Transcriptomic studies also indicate that a diversity of skin microbiota has more synergistic effects on host skin process such as epidermal proliferation, differentiation, and immune signaling than a single microbial species [104]. This highlights the necessity of the community-level interactions in the modulation of immune homeostasis and encouraging tissue repair [2, 102]. In the past, bacteria received a reputation as infection causes, but modern researchers observe their beneficial functions toward shaping immune responses that occur within the tissue and wound site. The communication between microscopic organisms and their human hosts happens primarily with immune receptors through microbial products and structural components from bacterial cell walls. Research shows SCFAs such as butyrate and acetate, which commensal bacteria generate, affect macrophage development toward M2 cells and help activate Tregs [102, 105]. Additionally, HDAC8 and HDAC9 inhibition by SCFAs in keratinocytes activates immune sensitivity toward MALP-2 along with poly(I:C), which leads to heightened IL-6 and IL-8 expression [102, 106]. Local differences emerge between different tissue types since microbial metabolites like SCFAs reduce monocyte inflammation but cause inflammatory reactions in keratinocytes, thus demonstrating microbe-derived metabolite responses depend on tissue context. The inflammation-regulating process through Toll-like receptor 2 (TLR2) is enabled by lipoteichoic acid (LTA), which is a component of Gram-positive bacterial cell walls [14]. Studies show that *S. epidermidis* activates γδ T cells present in human skin, which results in the production of P-2 bactericidal factor for enhanced antimicrobial protection combined with tissue homeostasis preserving mechanisms [2, 3, 107]. The antimicrobial and anti-inflammatory agent known as Reuterin is a product of *L. reuteri* metabolism, which also regulates the actions of macrophages. Interestingly, *S. epidermidis* generates tiny trace amine amounts, including tyramine and phenylethylamine, through its enzyme SadA [14, 108]. Studies demonstrated that TAs act as β2-adrenergic receptor antagonists, blocking the wound-suppressing effects of stress-induced adrenaline, thus promoting keratinocyte migration and accelerating wound healing [14]. The protein lactoferricin B performs functions similar to microorganisms by inhibiting inflammatory cytokines, including TNF-α and IL-6, and IL-8, and allows macrophages to assume an M2 phenotype [105, 109]. Through its action, microbiota shifts into a favorable composition that reduces *S. aureus*. Research confirms how wound therapy may benefit from microbiota-derived and inspired compounds to regulate immune responses in wounds.

The wound healing process accelerates via multiple host signaling pathways when skin commensal bacteria of the *S. epidermidis* strain participate [2, 3]. The best-studied mechanism occurs through the TLR system, according to research [14]. During skin injury, keratinocytes receive staphylococcal LTA that uses TLR2 to establish inhibitory signals, which mitigate TLR3 responses to maintain cellular homeostasis [14, 110]. The research by several studies explains how commensal-derived factors stimulate TLR2/EGFR/NF-κB signaling in keratinocytes to increase AMPs HBD-3 and RNase7 for microbial defense and skin healing [3, 14, 111]. The skin epithelial cells contain the aryl hydrocarbon receptor (AhR), which works as their main microbial detection system alongside other receptors [112, 113]. Keratinocytes receive signaling from *S. epidermidis* small-molecule ligands that activates AhR pathway signaling to induce IL-1β and IL-1α and CYP1A1 expression which strengthens immune response while improving barrier resilience [3]. Type I interferon (IFN-I) responses that stem from commensals have proven essential for early wound repair, according to the research [2, 114]. Besides interferon-mediated reactions commensal microbiota participates in epithelial regeneration by metabolic and transcriptional programming. Single-cell RNA sequencing and multiomic analysis revealed that the skin microbes, such as *S. epidermidis*, facilitate skin repair. They stimulate keratinocyte HIF-1α expression by glutamine metabolism, which is responsible for activating the hair follicle regeneration and skin reconstruction [115]. When skin-resident microbes activate neutrophils, the cells produce CXCL10 to attract plasmacytoid DCs (pDCs) to the site [2, 3]. The released interferon-α/β (IFN-α/β) from activated pDCs engages fibroblast and macrophage cells to generate essential growth factors VEGF and TGF-β for wound closure [2, 3, 116]. Both bacterial signaling pathways operating with host immune responses and skin cells enable an effective interlock that accelerates overall wound recovery and decreases healing scarring.

### Probiotic applications in wound healing via immunoregulation

The initial knowledge about how commensal and probiotic microbes impact immune system regulation in wound healing allows us to move forward to explore specific probiotic strains that consistently regulate immune responses in skin trauma recovery. The immune system functions as a central regulator during wound resolution, but scientists now see the use of probiotics to adjust immune environmental signals may be as an alternative therapeutic approach.

The immunomodulatory properties of skin wound healing along with therapeutic capacity were investigated using seven widely researched probiotic strains consisting of *Lactobacillus plantarum* (*L. plantarum*), *Lactobacillus rhamnosus* (*L. rhamnosus*), *L. reuteri*, *Lactobacillus casei* (*L. casei*), *Lactobacillus acidophilus* (*L. acidophilus*), *Bifidobacterium longum* (*B. longum*), and *Bifidobacterium bifidum* (*B. bifidum*). Selection activities for the presented studies in Tables 2, 3, 5, and  6 were conducted thoroughly and rigorously. The PubMed database search using seven keyword combinations produced results of 49, 28, 36, 27, 19, 7, and 2 for the terms ‘*L. plantarum*, wound healing’ and ‘*L. rhamnosus*, wound healing’ and ‘*L. reuteri*, wound healing’ and ‘*L. casei*, wound healing’ and ‘*L. acidophilus*, wound healing’ and ‘*B. longum*, wound healing’ and ‘*B. bifidum*, wound healing’. The screening process moved forward to identify studies dedicated to researching skin wound healing effects of these probiotics. As a result, 35, 19, 25, 16, 4, 6, and 1 articles were identified for *L. plantarum*, *L. rhamnosus*, *L. reuteri*, *L. casei*, *L. acidophilus*, *B. longum*, and *B. bifidum*, respectively. An additional layer of manual review was conducted: two authors independently evaluated and double-confirmed the inclusion of original studies dedicated to exploring the immunoregulatory effects of probiotics in the context of skin wound healing. The selected research data appear in Tables 2 and 3 for immunological effects of different probiotic strains, and Tables 5 and 6 provide evidence on engineered probiotics used for wound healing applications.

**Table 2:** Immunoregulatory mechanisms of probiotics and their effects on wound healing outcomes

Category	Probiotics	Effects	Outcomes	References
Macrophage modulation (M1/M2)	*L. plantarum*, *Lactobacillus rhamnosus*, *L. reuteri*, *Lactobacillus casei*, *Bifidobacterium animalis*	Promotes M2 phenotype, reduces inflammation, enhances angiogenesis	Accelerated healing, scarless tissue regeneration, improved angiogenesis (↑CD31^+^ vessels, ↑α-SMA^+^ myofibroblasts)	[117–122]
Treg modulation	*L. reuteri*	Enhances Treg activity, oxytocin-linked systemic response	Faster epithelialization, systemic Treg response, improved immune tolerance	[123]
Cytokine regulation	*L. plantarum*, *L. rhamnosus*, *Lactobacillus reuteri*, *L. casei*, *Lactobacillus acidophilus*, *Lactobacillus salivarius*, *Lactobacillus gasseri*, *Lactobacillus paracasei*, *L. bulgaricus*, *Bifidobacterium longum*, *Bifidobacterium lactis*	↓ TNF-α, IL-6, IL-1β, mmp-9; ↑ IL-10, TGF-β, VEGF	Decreased inflammation, faster epithelialization, reduced scarring, better collagen remodeling, wound size reduction	[124–140]
Chemokine regulation	*L. plantarum*, *L. rhamnosus*, *L. casei*, *L. acidophilus*, *B. lactis*, *Bifidobacterium pseudolongum*	Regulates IL-8; ↑ CXCL2/CXCR2 axis, CXCL8-CXCR1/2 axis, CXCL10; ↓ CXCL2, CXCL6, CXCL8	Faster keratinocyte migration, reduced neutrophil over-recruitment, improved epithelial closure	[141–145]

↑: increase; ↓: decrease; *α-SMA* alpha smooth muscle actin, *CXCL* C-X-C motif chemokine ligand, *CXCR* C-X-C motif chemokine receptor, *IL* interleukin, *IL-8* interleukin 8, *CXCL8* C-X-C motif chemokine ligand 8, *MMP-9* matrix metalloproteinase-9, *M1* pro-inflammatory M1 macrophage, *M2* anti-inflammatory M2 macrophage

**Table 3:** Microenvironmental and molecular signaling modulation by probiotics in wound healing

Functions	Probiotics	Mechanisms	Effects	Outcomes	References
Antioxidant modulation	*L. plantarum*, *Lactobacillus reuteri*, *Lactobacillus casei*	↑ NOS2 and GSH/catalase; ↑ VEGF/TGF-β	↓ ROS, MMP-9, TNF-a; ↑ Angiogenic	Faster re-epithelialization, reduced oxidative stress and inflammation, improved healing in diabetic and burn models	[146–150]
Tropic modulation	*L. plantarum*, *L. reuteri*	↑ TGF-β, VEGF, FGF, oxytocin ↓ Corticosterone	↑ Anti-stress, pro-angiogenic	Improved wound closure under stress, systemic repair effects (oxytocin-linked)	[151, 152]
Quorum sensing modulation	*L. plantarum*, *L. reuteri*, *L. casei*, *Lactobacillus fermentum*, *Bifidobacterium longum*, *B. subtilis*	Quorum sensing inhibition, AI-2 interference; ↓ Virulence; ↑ AMPs (β-defensin 1), cathepsins B/D/H	↓ Biofilm; ↓ Inflammation	Improved healing speed, lower infection rates, better microbial balance	[151–160]
Signaling modulation	*L. plantarum*, *L. rhamnosus*, *L. reuteri*, *L. casei*	↑ Wnt signaling, FAK/AKT activation, FAK-independent p38 and ERK phosphorylation, PI3K/AKT axis, HIF-1α signaling, TGF-β signaling, TLR2/MAPK axis, TIMP1/2,; ↓MMP1/2/3/9/10, collagenase, elastase	↑ Regeneration, proliferation, antioxidant protection; ↓ Inflammation	Enhanced epithelialization, angiogenesis, keratinocyte/fibroblast migration, ECM remodeling, improved microbiota balance, strain-specific immunomodulation	[161–167]

↑: increase; ↓: decrease; *AKT* protein kinase B, *ECM* extracellular matrix, *ERK* extracellular signal-regulated kinases, *FGF* fibroblast growth factor, *FAK* focal adhesion kinase, *GSH* glutathione, *HIF* hypoxia-inducible factors, *MMP* matrix metalloproteinase, *MAPK* mitogen-activated protein kinase, *NOS2* nitric oxide synthase 2, *PI3K* phosphoinositide 3-kinase, *TLR2* toll-like receptor 2, *TIMP* tissue inhibitors of metalloproteinases

#### Animal studies on probiotic-mediated immunomodulatory wound healing

Research findings demonstrate that specific *Lactobacillus* and *Bifidobacterium* strains gain substantial interest because they boost wound recovery by controlling immune functions directly and indirectly. These include attenuation of pro-inflammatory cytokines such as TNF-α, IL-6, IL-1β, promotion of anti-inflammatory and tissue-repairing mediators such as IL-10, TGF-β, VEGF, and polarization of macrophages toward the M2 phenotype [117, 118, 124–128]. The beneficial strains make their impact felt through two pathways that work at both a systemic immune level and through direct local interactions within a wound. Three derivatives from these probiotic strains, including lysates, extracellular vesicles, and secreted metabolites, are capable of influencing epithelial cell migration, angiogenesis, collagen remodeling, and biofilm disruption. Research shows *L. plantarum* and *L. rhamnosus* improve keratinocyte activity and reduce inflammasome activation effectively but *L. reuteri* and *L. casei* regulate Treg cells and reduce oxidative stress [123, 125, 129, 141, 146, 151, 153, 154, 161]. Furthermore, *L. acidophilus* and *B. longum* deliver therapeutic benefits through topical and systemic treatments by controlling immune cell traffic and enhancing fibroblast proliferation and modifying neurocutaneous immune function [130, 142]. The following segment presents an organized summary of six significant probiotic strains that demonstrate wound healing ability for the skin through modulation of immune responses and their associated therapeutic benefits. Existing evidence regarding preclinical and clinical applications appears in Tables 2 to 4, which organize data through model system evaluations and immune system interactions, as well as major wound healing results. Through data consolidation, this research seeks to show the wide microbial effects while finding common routes of immune response affected by various probiotic agents.

**Table 4:** Summary of NCT-registered trials investigating probiotic therapies for various wound types

Trial ID	Target condition	Design	Intervention	Endpoint	Preliminary outcome
NCT05608187	Diabetic foot ulcer	Randomized, double-blind, placebo-controlled, parallel, exploratory phase 2a	Low dose of ILP100-Topical (engineered *L. reuteri* expressing CXCL12) (5 × 10^7^ CFU/cm^2^) High dose of ILP100-Topical (1 × 10^9^ CFU/cm^2^) Placebo	Safety, tolerability, and wound healing efficacy	Terminated due to patient recruitment issues
NCT04281992 [44]	Diabetic foot ulcer	Non-randomized, uncontrolled Phase 1 and randomized, placebo-controlled Phase 2	Single dose of AUP1602-C (engineered *Lactococcus cremoris* expressing FGF-2, IL-4, CSF-1) (2.5 × 10^5^ CFU/cm^2^) Repeated doses between 2.5 × 10^6^ and 2.5 × 10^8^ CFU/cm^2^ administered 3 times per week for 6 weeks	Safety, tolerability, and wound healing efficacy	AUP1602-C was safe, well tolerated, and achieved 83% complete healing in non-healing diabetic foot ulcer patients at the recommended phase II dose, supporting further clinical development
NCT02572531 [183]	Post-extraction oral wound	Randomized, double-blind, placebo-controlled trial	*L. reuteri* DSM 17938 and ATCC PTA 5289: three lozenges per day for two weeks Placebo	Wound healing efficacy and extra-oral swelling	Notable patient-perceived improvements in post-operative recovery with *L. reuteri* lozenges supplementation, despite limited significant changes in objective wound healing outcomes
NCT04903925	Post-extraction oral wound	Randomized, double-blind, placebo-controlled trial	Probiotic *L. reuteri* for 21 days (2 tablets/day) and amoxicillin antibiotic therapy with for 6 days (2 g/day) Placebo	Wound healing efficacy	Not applicable due to recruiting
NCT03210779 [184]	Oral mucosal wound	Randomized, double-blind, pilot study	*L. reuteri* DSM 17938 and ATCC PTA 5289: lozenges (≥5 × 10^8^ CFU/strain, 3×/day) + probiotic oil (2 × 10^8^ CFU/ml, 1×/day) for 8 days Placebo	Expression of MMPs and interferons in wound exudate	MMP and interferon (IFN-α2, β, γ) levels decreased over time during probiotic *L. reuteri* treatment, but differences versus placebo were not statistically significant
NCT06674564	Wound healing in laryngeal cancer patients post-laryngectomy	Randomized, open-label, parallel study	Probiotics and zinc group Probiotics group Zinc group (Zinc Acetate 50 Mg Oral Capsule) Control group	Wound healing, infection rates, occurrence of percutaneous fistula, starting of oral feeding	The study is ongoing, and participants are receiving an intervention or being examined, but potential participants are not currently being recruited or enrolled

*CSF* colony-stimulating factor, *CFU* colony-forming unit, *CXCL* C-X-C motif chemokine ligand, *FGF* fibroblast growth factor, *IL* interleukin, *IFN* interferon, *MMP* matrix metalloproteinase

(i) Evidence shows *L. plantarum* produces major immunomodulatory changes in skin wound healing through *in vitro* and *in vivo* studies. Through its regulatory function, *L. plantarum* controls both cytokine and signaling pathway activities in the local immune environment. Scientific studies reveal that *L. plantarum* exerts anti-inflammatory properties by reducing TNF-α, IL-1β, and IL-6 but increasing IL-10, TGF-β, VEGF, and CXCL2 production [119, 125, 143, 155, 185, 186]. The regulatory effects of these actions help decrease neutrophil recruitment while simultaneously inhibiting NLRP3 inflammasome activation to suppress cell death pathways and promote M2 macrophage differentiation [119, 131]. Moreover, the derivatives of *L. plantarum*, including lysates and supernatants together with extracellular vesicles, demonstrate their ability to diminish pathogenic quorum sensing and bacterial biofilm formation while reducing pathogen population to reduce persistent inflammation according to existing research [120, 147, 156, 157]. The immunomodulatory actions of these interventions support wound healing by establishing the proper transition between inflammation and proliferation, and enable re-epithelialization and collagen remodeling processes, which lead to improvements in wound closure alongside scar reduction [120, 148]. (ii) *L. rhamnosus* strain GG (LGG) shows extensive immunomodulatory effects on the skin healing process. The LGG strain affects both nearby and broader immune responses by multiple mechanisms to drive tissue repair. The anti-inflammatory changes promoted by LGG reduce TNF-α and IL-6, whereas supporting M2-polarized macrophages and regulating CXCL2/CXCR2 keratinocyte signaling to promote cell migration during wound healing [125, 143, 187]. LGG reinstates disrupted antibiotic-impaired immune balance by switching on the Wnt/β-catenin and AKT/FAK regenerative mechanisms [162, 163]. The extracellular vesicles from LGG tissue have demonstrated their ability to promote angiogenesis along with re-epithelialization through the regulation of miR-21-5p [164]. The intake of *L. rhamnosus* through the oral route leads to better wound healing results because it strengthens the gut–skin immune connection, together with gut-associated lymphoid tissue function enhancement [132, 188]. Laboratory research, along with clinical trials, proves this remedy effective for tissue reduction and swelling and scar tissue prevention, which presents potential benefits in current wound treatments [117, 127, 129]. (iii) *L. reuteri* plays a pivotal role in modulating the immune microenvironment to enhance skin wound healing. Wound repair experiences a transition to pro-regenerative phases because *L. reuteri* activates multiple key immune cell activities according to documented research findings. Given exposure to *L. reuteri*, the immune cells known as macrophages switch their behavior to an M2 phenotype, an anti-inflammatory type, while additional TGF-β and IL-10 regulatory proteins develop locally, and the pro-inflammatory agents TNF-α and IL-6 decrease in production. Cellular immune cell recruitment and tissue remodeling get improved through engineered strains that express chemokines like CXCL12. The gut-brain-immune axis network activates Tregs through oxytocin production caused by *L. reuteri* edible to create a system-wide healing-promoting environment [189]. The immunomodulatory capabilities of *L. reuteri* advance wound repair and minimize infections while promoting tissue restoration making it a suitable microbiome-based therapeutic choice for wounds [43, 45–47, 118, 121, 123, 133, 134, 143, 149, 151, 165, 166, 190–192]. (iv) Research has proven ***L. casei*** to be an effective biological agent that promotes skin wound healing through different immunomodulatory mechanisms. The tissue regenerative properties of *L. casei* and its derivatives, including live cells and paraprobiotics along with membrane vesicles (MVs) and exopolysaccharides (EPS), and cell-free supernatants, have consistently exhibited immune response modulation both within innate and adaptive immunity through *in vitro* and *in vivo* and clinical research platforms [128, 135, 144]. Living *L. casei* and its derivatives can transform macrophage polarization patterns from M1 inflammatory to M2 healing phenotype, which leads to decreased inflammation while supporting tissue restoration [120, 122]. The pro-inflammatory cytokines TNF-α and IL-6, together with IL-1β, decrease in response to *L. casei* compounds, while chemokines CXCL8 and their receptors (CXCR1/2) experience increased production, aiding epithelial cell migration and angiogenesis processes [128, 144]. Extracellular polysaccharides derived from *L. casei* suppress the expression of matrix metalloproteinases (MMPs) and thus help maintain dermal ECM structure to drive dermal remodeling [167]. *L. casei* produces immunomodulating actions that include antimicrobial activity against resistant pathogens and biofilm destruction and anti-stress effects against oxidative damage [136, 158]. Stem cell preconditioning with *L. casei* metabolites results in three types of beneficial effects on cells when placed in the wound area: increased survival, together with enhanced antioxidant functions and antimicrobial capabilities [146, 158]. (v) *L. acidophilus* has demonstrated significant immunomodulatory effects that contribute to skin wound healing. Stimulated immune responses by *L. acidophilus* cause production of cytokines IL-1β and IL-10 and IFN-α and TNF-α that are vital for triggering and regulating wound healing processes [130]. The activation and proliferation of T-lymphocytes CD4^+^ cells, natural killer cells, and macrophages become active while supporting both angiogenesis and fibroblast activity. Research indicates that *L. acidophilus* regulates chemokine release from human intestinal subepithelial myofibroblasts by decreasing CXCL2, CXCL6, and CXCL8 while enhancing CCL10 signaling pathways responsible for immune cell migration and tissue reorganization [142]. (vi) *B. longum* exerts significant immunomodulatory effects in the context of skin wound healing. *B. longum* produces topical and cell-based preparations that promote epidermal differentiation and boost AMPs production of β-defensin 1 while enhancing the expression of cathepsins B, D, and H—these enzymes participate in tissue remodeling alongside host protection [159]. The bacterial formulation demonstrates its role in controlling inflammatory processes by decreasing skin sensitivity, together with its effect on inhibiting inflammation triggered by neuropeptide activity in keratinocytes. Research using *in vitro* experiments showed that *B. longum* lysate soluble fractions protected keratinocytes from cell death, while blocking NOS2 activity demonstrated that bacterial healing functions proceed through NO biological pathways [147]. Research conducted on laboratory animals demonstrated that *B. longum* used together with *L. rhamnosus* stimulated angiogenesis and fibroblast activation during the wound healing process which enhanced tissue remodeling and granulation [141, 191]. A systematic review indicated that *B. longum* provides multi-stage immune regulatory benefits that target both inflammatory control and cell recovery, and blood vessel development as a part of multi-strain probiotic combinations [191]. The combined effects lead to an immune-stable conditioning of the wound area that accelerates skin regeneration without creating infections or scarring.

#### Clinical studies on probiotic-mediated wound healing

The immunomodulatory and pro-healing benefits of probiotic strains are now being confirmed through emerging human clinical trials involving various wound healing situations. The results from multiple studies demonstrate that probiotic-based treatments hold strong clinical value for regulating immune responses to facilitate the healing of wounds. A triple-blind randomized controlled trial on primiparous women consuming *L. casei* 431 through oral supplements showed the strain enhanced episiotomy wound healing during a 15-day period based on decreased redness scores and reduced edema and discharge amounts [140]. Medical studies showed that the strain produces both systemic anti-inflammatory properties and restores mucosal immune equilibrium. Similarly, the topical application of *L. plantarum* cultures on 34 patients led to wound debridement and granulation tissue development within 10 days while simultaneously altering neutrophil IL-8 productions and necrotic cell count reduction at the ulcer site [145]. The administration of probiotics initiates a cytokine shift toward day 5 that leads to a reparative immune environment and eventually reduces at day 10. Treatment of burn patients with topical *L. plantarum* bacteriotherapy represents another clinical application. Studies have shown that the application of *L. plantarum* results in equivalent or superior results than silver sulfadiazine when it comes to bacterial reduction and granulation tissue formation in both second- and third-degree burns [155]. Evidence suggests that topical *L. plantarum* bacteria-free supernatant shows safety properties and reduces burn wound infection and transplant rejection in deep second-degree burns, thus offering promise for wound healing treatment [193]. Research on pediatric burn patients through a randomized controlled trial proved that consuming probiotics orally remained safe and showed promising results to reduce fungal infections while speeding up wound healing particularly in a clinically vulnerable population [188]. Additionally, a randomized controlled trial found that applying *L. reuteri* through lozenges and topical oil to the mouth helped speed up early wound healing in the mouth while showing promising changes in nearby cytokines despite falling short of statistical proof [194]. The approach minimized the need for conventional antimicrobial use thus maintaining beneficial immune-modulating outcomes that promote tissue healing. In the oral cavity, two clinical trials further confirmed the immunotherapeutic potential of probiotics. The pilot study NCT02572531 reported notable patient-perceived improvements in post-operative recovery with *L. reuteri* lozenges supplementation, despite limited significant changes in objective wound healing outcomes [183]. Additionally, another research study (NCT03210779) suggested that MMP and interferon (IFN-α2, β, γ) levels decreased over time during probiotic *L. reuteri* treatment, but differences versus placebo were not statistically significant, yet additional extensive investigations must confirm these preliminary results [184].

To further consolidate emerging clinical evidence and to capture the existing research scenario, we were able to systematically search the ClinicalTrials.gov database to build a table with a summary of all its clinical trials. Specific intervention keywords were used during the search, which are eight probiotic strains, i.e. *L. plantarum*, *L. rhamnosus*, *L. reuteri*, *L. casei*, *L. acidophilus*, *B. longum*, *B. bifidum*, and *L. cremoris*. In the condition field, it was used wound, burn, or skin terms to search trials that assess the therapeutic properties of probiotics at various wound types. Appropriate studies were screened on the basis of wound healing data, and they were enrolled irrespective of recruitment status. Published results were also consulted, where possible and consequently included in the analysis. The six selected studies are summarized in Table 4, showing an overview of the ClinicalTrials.gov (NCT) posted trials that explore the probiotic therapy of different types of wounds. Based on the summarized trials, it can be said that some patient particularities may affect the effectiveness of probiotic interventions. Various physiological dysfunctions in diabetic patients drastically change the microenvironment of the wounds, which may change the success of measures based on probiotics. Chronic hyperglycemia affects the performance of the leukocyte, slows collagen production, and maintains the state of low-grade inflammation, whereas diabetic neuropathy suppresses wound perception and causes a delay in wound treatment [195–197]. In addition, microvascular complications restrict the dispersion of oxygen and nutrients, which reduces tissue regrowth further [198, 199]. Microbiome in DFU is usually dysbiotic, and heavy loads of pathogenic bacteria are present, which may compete with beneficial probiotics in niche colonization [200, 201]. Remarkably, the non-healing nature of DFU (nhDFU) indicates the existence of pathophysiological conditions of dysregulated immune response that support the sooner introduction of systemic or repeated probiotic use combined with individual topical administration. Phase I study of AUP 1602-C also revealed that older age (53–80 years), chronicity of the wound, depth of the wound, and history of previous treatment failure are among the key modifiers of response, perhaps because of age-related decline of angiogenesis, fibroblast function, and epithelial regeneration capability [44]. Therefore, a thoroughly probiotic approach may hold promise to regulate both systemic and local alterations of the immunometabolism in diabetic wounds. Moreover, burn wounds have a specific clinical characteristic as they are accompanied by acute tissue injury, overwhelming inflammation, and a strong likelihood of colonization with multidrug-resistant (MDR) pathogens, including *Pseudomonas aeruginosa* [202–204]. Localized applications will be especially pertinent as a damaged epithelial barrier allows direct contact between the topical probiotics and wound tissue. During the initial phases, burn wounds are accompanied by a vigorous yet haphazard immune response, frequently shifting toward what is referred to as immunoparalysis where the ability of the host to combat infection and induce repair is impaired [205]. Topical use of *L. plantarum* (either as live culture or bacteria-free supernatant) has demonstrated positive results on the reduction of the bacterial burden and enhancement of granulation tissue formation, in some aspects even exceeding silver sulfadiazine in clinical parameters [155, 193]. Nonetheless, the results of treatment may be changed by the individual characteristics, including burn depth, wound size, the existence of infection, and the host microbiota composition. Activity and colonization of probiotics may be influenced by the presence of necrotic tissue or dryness and pre-exposure to antibiotics. Hence, the probiotic techniques used in burn-related situations should address both the microbial setting of the wound and the immunological stage to maximize their efficiency.

Taken together, the clinical data fully support the results from *in vitro* and animal model research to demonstrate that probiotics perform active management of immune responses beyond simple bacterial colonization of wounds. These probiotics support tissue repair through their functions in cytokine regulation and neutrophil recruitment control and regulatory T cell response enhancement. As such, clinical application of probiotics in wounds after surgery and chronic ulcers and burns appears to offer a valuable addition to conventional antimicrobial treatments and anti-inflammatory methods. The data demonstrate that probiotic agents take an active role alongside host cells for regulating immune functions throughout trauma healing processes. Therapeutic development needs rethinking because instead of trying to eliminate all bacteria, researchers should develop methods to manage microbial activities within wounds for optimized results. In fact, the immunoregulatory properties of probiotics create a basis for developing engineered probiotic products as future therapeutic options. Natural probiotic strains display capabilities to modify macrophage actions while activating Tregs and facilitating wound skin growth, which creates the foundation to create engineered probiotics that deliver specific cytokines or anti-inflammatory compounds in a controlled manner. For example, engineered probiotic strains designed to release IL-10 and VEGF as an appropriate response to wound signals would optimize healing yet remain localized to wound sites. As such, knowledge about how probiotics modulate immune response in their natural settings enables researchers to grasp universal wound complexity and design effective engineered therapeutic solutions.

### Engineered strategies for probiotic modulation

#### Engineered probiotics: a targeted approach to complex healing

Treatment with conventional drugs proves insufficient for dealing with the complex requirements of problematic environments that arise in chronic inflammatory and ulcerative wounds. The main limitations that affect traditional drugs include their brief survival duration in the body, combined with reduced efficiency under unfavorable environmental conditions [206–209]. Chemical drugs quickly break down under GI conditions of acidity and enzymes, yet biologic therapies need multiple high dosages to work properly because they harm more parts of the body, exhibit off-target effects [208, 209]. In contrast, the use of engineered probiotics presents itself as a different strategy to existing problem. The living therapeutics exhibit biological compatibility and motility together with colonization ability, which allows them to interact accurately and maintain long-term contact with target tissues [210]. Genetic or surface engineering increases the capabilities of probiotics to function as programmable drug factories that deliver therapeutic agents with spatial and temporal control [211, 212]. Local therapeutic approaches, along with reduced systemic toxicity, become achievable through this strategy, which improves hard-to-treat skin treatment outcomes during chronic wound recovery and drug delivery challenges and immune deficiency states. Self-replication among engineered probiotics provides a unique advantage because the substances they carry achieve increasing therapeutic potency through natural replication while eliminating the requirement for additional doses. Through their metabolic flexibility and their ability to interact with host microorganisms, they achieve combined therapeutic benefits such as immune response management and barrier health improvement and microbial community reestablishment [31].

#### Genetic engineering: probiotic platforms for skin wound healing

The application of genetic engineering to probiotics created opportunities for treating skin wounds, including difficult-to-heal and infected and inflammatory wounds. The efficacy of traditional treatments suffers from three major limitations that including factor degradation upon application and a broad focus area, along with systemic adverse effects. In contrast, engineered probiotics serve as intelligent micro-factories that enter wound beds to perform local signal-based treatments through therapeutic molecule production.

##### CRISPR-Cas9: the precision engine of synthetic probiotics

Clustered regularly interspaced short palindromic repeats-associated protein 9 (CRISPR-Cas9) is a formidable genetic editing technology, which was initially found in bacterial immune system, protecting them against viruses [213, 214]. It was later transferred into one of the most accurate and dynamic tools of engineering microorganisms [215–217]. Applied to synthetic probiotics, CRISPR gives scientists the chance to re-engineer good bacteria such as *Lactobacillus* or engineered *E. coli* to undertake a given therapeutic action in the body. The past developments in technology allowed the CRISPR-based tools to not only become more accurate, but also safer and easier to use in living organisms. An example is given through the recent introduction of novel split-CRISPR systems that make it possible to design temporally-regulated RNA transcription, without persistent expression, thereby improving *in vivo* safety [218]. The system allows the turning on or off of genes only when necessary similar to a light switch activated by a signal. Such control minimizes the unwanted impact and maximizes safety with regard to applying probiotics to humans. Further, ultra-compact Cas12f and improved variants of Cas9 with extended PAM specificity have facilitated effective delivery using viral vectors to make *in vivo* microbiota editing [219]. The packaging of these proteins into delivery systems (such as viral vectors), which may be targeted toward microbes already present in the gut or skin is now more straightforward. Besides, it has been demonstrated that prime editing is a new generation, precision-editing, permitting a versatile range of base changes and small insertions or deletions even without the creation of double-stranded breaks, and it has potential in the manipulation of both host and microbial genomes [220]. The method promises even greater accuracy because it modifies genetic information without severing DNA in a brutal way similar to changing just one word in a paragraph without ripping the entire paper. Also, CRISPR-associated transposase systems (CASTs) were recently modified to integrate genomes in bacteria, including the gut, in a stable and multiplexed manner [221]. This method will allow inserting many genes into the genome simultaneously, and it is possible to construct larger and more stable synthetic circuits inside the bacteria. Collectively, these developments are making CRISPR a tool for constructing smart probiotics-engineered bacteria that can sense their environments, adapt to changing environments, and be able to perform specific medical duties [222–224].

**Figure 3:**
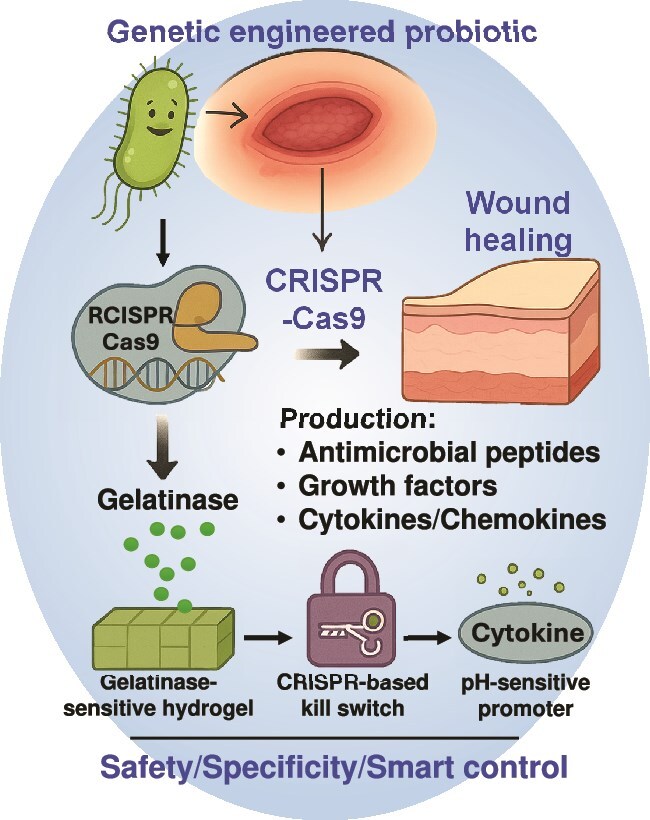
Schematic model of CRISPR-Cas9–engineered probiotics for targeted wound healing. The figure depicts that the CRISPR-Cas9-programmed genetically modified probiotics produce the therapeutic agents circulating throughout the whole body via regulated release of therapeutic factors such as the antimicrobial peptidic factors, growth factors, and cytokines in response to the wound-specific signals such as pH, ROS, and bacterial density. An elucidated role of this system is the involved biosafety mechanism of using CRISPR-based killing switches and gelatinase-sensitive hydrogels to increase therapeutic specificity, spatiotemporal control, and safety in the context of inflamed-infected wound conditions. *CRISPR* clustered regularly interspaced short palindromic repeats, *Cas9* CRISPR-associated protein 9

In wound healing applications, (i) The CRISPR system permits targeted gene elimination through deletion of virulence factors like the removal of msbB or LPS genes in Salmonella or *E. coli* bacteria for creating safer clinical strains [210]. (ii) The CRISPR system provides the capability to introduce therapeutic features into cells through insertion of EGF, VEGF growth factors and IL-10 anti-inflammatory cytokines [225]. (iii) CRISPR enables developers to build promoters that activate gene expression upon detection of specific wound triggers, including low oxygen and high ROS and acidic pH. (iv) CRISPR enables the development of multilayered gene circuits that use CRISPR logic gates to enable probiotics to determine when to produce antibiotics and enzymes, as well as regenerative molecules, through sensing quorum signals or host indicators. The design of probiotics utilizing CRISPR-controlled circuits allows them to produce epidermal growth factor (EGF) in hypoxic wound conditions while avoiding systemic exposure [225, 226]. Similarly, probiotics may be designed to be modified with CRISPR-activated EGF secretion modules to enhance neovascularization in ischemic wounds [227, 228]. Figure 3 provides a conceptual design of the genetically engineered probiotics adapted for wound healing applications that incorporate genetic control, environmental sensitivity, and safety mechanisms in a unified programmable design. The schematic concept of how probiotics can be engineered using the systems of the CRISPR-Cas9 system to produce wound-relevant therapeutic products, including cytokines, growth factors, and AMPs under precise transcriptional control. The option of responsive promoters, such as pH-sensitive elements, provides the opportunity to activate therapy in a localized way, i.e. specifically in the acidic microenvironment of the chronic wound. In parallel, in order to make sure that the engineered probiotics only work at the site of the desired wound, the design incorporates a gelatinase-sensitive hydrogel delivery system. This hydrogel matrix encapsulates the probiotic strains and does not break down under normal circumstances, but will break down, reacting with gelatinase enzymes (usually elevated in chronic wounds). Upon breakdown by the enzymes, the probiotics are selectively released to the wound microenvironment. This allows for local distribution and avoids systemic distribution which is in favor of tissue-specific action and reduction of potential off-target effects. Moreover, a biosafety guarantee of a kill switch is also embedded via CRISPR to avoid long-term colonization or horizontal gene transfer to solve the regulatory and ecological issues in live microbial therapeutics. This figure represents a step forward in the field in moving beyond this generalized probiotic engineering to propose a next generation, modular design framework in which gene circuits can be dynamically controlled according to wound microenvironment cues. It represents a transition toward smart therapeutics, responsive, programmed, and controlled microbial systems that have a potential for precision therapy at the site of the injury. Such designs are not only technologically feasible, with current tools of synthetic biology, but they are a blueprint for future clinical translation, which emphasizes the importance of safety, controllability, and target specificity. As illustrated in the figure, the combination of environmental sensors, safety switches and tissue-adaptive delivery systems into a single microbial chassis may potentially provide a significant boost for the implementation of engineered probiotics into wound care.

##### Engineered therapeutic functions for wound care

Probiotics can gain specific therapeutic properties using genetic engineering and synthetic biology that allow probiotics to gain specific properties. Overall, the following three categories of the engineered functions may be distinguished: antimicrobial effects, tissue regeneration through growth factors, and inflammatory response control through cytokines.

###### Antimicrobial peptide delivery

The engineering design of probiotics to produce various peptides like LL-37, nisin, and CRISPR-guided bacteriophage-like agents for targeting *S. aureus* and *P. aeruginosa* biofilms [229–234]. These are two pathogens that are usually linked with chronic wound infection. Engineered probiotics are capable of circumventing resistance by local and sustained administration of antimicrobial agents and decreasing the microbial burden of wounds.

###### Growth factor secretion

**Figure 4:**
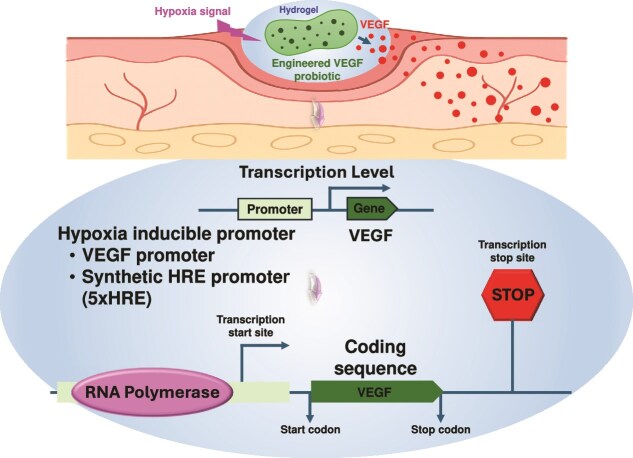
Hypoxia-inducible VEGF expression in engineered probiotics for wound vascularization. This figure shows a schematic illustration of how engineered probiotics in capsules of hydrogel respond to a hypoxic wound signal in activating the production of VEGF through the activation of hypoxia-inducible promoters such as the VEGF promoter or synthetic 5×HRE promoter. Such a design utilizes HRE-mediated transcriptional activation of HIF-1α binding that ensures site-specific and stimulus-responsive delivery of VEGF therapeutic agents to improve angiogenesis of ischemic wounds

The border of skin healing research explores ways to boost tissue regeneration together with the process of angiogenesis. Probiotics may be designed to be engineered to produce and secrete EGF, VEGF, and PDGF [31]. Probiotics also may be designed to be modified with a plasmid encoding EGF under hypoxia-inducible promoter can be applied to ischemic wounds to locally boost keratinocyte proliferation and re-epithelialization [235]. Probiotics may be designed to be engineered to secrete VEGF improves capillary density and oxygenation in diabetic wounds [236]*.* As shown in Figure 4, engineered probiotics which are implanted within hydrogels, can use the hypoxia-sensitive promoter to express VEGF locally, in response to a local ischemic stimulus, enhancing vascularization at the wound site in a spatially regulated way. Encapsulated inside biocompatible hydrogels, these engineered strains stay spatially confined in the wound site where they induce the expression of the antiangiogenic protein, the VEGF, only in hypoxic conditions, which serves as a hallmark for a poorly vascularized chronic wound. To regulate gene expression at low oxygen tension, hypoxia-inducible promoters that are commonly relevant are the native VEGF promoter with an 1xhypoxia hypoxia-responsive elements (HREs) and synthetic constructs with 5×HRE [237–239]. HREs activate such systems and bind the hypoxia-inducible factor (HIF-1α) in situations of ischemia. Other characteristics of these systems are oxygen sensitivity. The transcription process activity is suppressed at normal oxygen (normoxia) and activated in a hypoxic condition, where genes can be controlled spatially and temporally. An early study of this revealed that VEGF expression and pathological neovascularization were dramatically lowered in mouse models of ischemic retinopathy and choroidal neovascularization by deletion of the HRE in the VEGF promoter and thus established the importance of the HRE in the induction of VEGF during hypoxia [237]. By the same token, other research also established that gene delivery of VEGF under the control of a synthetic promoter consisting of five tandem repeats of hypoxia-responsive element (5×HRE) specific to collagen matrices produced significant neovascularization and cardiac functional recovery after myocardial infarction, highlighting the translatability of 5×HRE-based promoters to ischemic targeted therapeutic agents [239]. The transcriptional regulation mechanism is shown in detail in the figure with both native and artificial promoter options. Specifically, it focuses on the use of hypoxia-inducible promoters such as the native promoter for the protein VEGF containing 1×HRE (hypoxia responsive element) and a synthetic 5×HRE construct, which respond to (low) oxygen tension through binding to HIF-1α. The design contains the transcriptional start and stop codons, promoter regions and the well-defined coding sequence highlighting the modularity and synthetics of gene expression under hypoxia. This programmable expression system is spatially/temporally precise and does not run the risks of off-target effects and overexpression during normoxic conditions. By confining the developed engineered probiotic in a gelatinous hydrogel, the existence makes sure that the probiotic could have a localized retention time, a longer residence time, and controlled interaction with the ischemic tissue. Beyond the conceptual novelty, this design has some critical implications to the field of regenerative medicine. Compared to bolus injections of a recombinant form of the protein, for instance, which has short half-lives and systemic side effects, this process offers a sustained, localized and wound microenvironment-dependent therapeutic effect. It also shows how synthetic biology tools, such as promoter engineering, can be used to create environment-responsive gene circuits for precision medicine. The success of 5×HRE-based promoters in other ischemic models, including myocardial infarction and retinopathy, also provides further evidence for the translatability of such a strategy to chronic wound care, where impaired vascularization is one of the barriers to healing. This figure thus provides a bridge between the synthetic control of genes and the pathophysiology of a wound, providing a conceptual framework for the development of biotherapy strategies for chronic and non-healing wounds.

###### Cytokine secretion

Chronic wounds are often arrested in the inflammatory phase. The balance between pro- and anti-inflammatory cytokines can be restored through genetically modified probiotics that produce anti-inflammatory agents. Engineered probiotics may be built specifically to produce the anti-inflammatory cytokine IL-10, which helps decrease inflammatory cytokines TNF-α and IL-6 in wound models [31, 240–243]. Studies showed that the genetic modification of probiotics to generate anti-inflammatory cytokines including IL-10 or IL-35, successfully regulates inflammatory bowel disease (IBD) along with enhancing the restoration of immune equilibrium [32, 244–250]. However, anti-inflammatory engineered probiotics remain insufficiently investigated for skin wound healing although their future application shows great promise for treatment development. Macrophage polarization toward the M2 phenotype emerges through the use of probiotics contained in hydrogel scaffolds as they enhance wound bed tissue remodeling and collagen formation. These immune-modulatory probiotics function best for treating diabetic wounds or pressure ulcers because immune system management allows proper wound healing.

##### Safety, specificity, and smart control

The field now utilizes synthetic gene circuits to develop therapeutic *smart probiotics* which activate therapeutics production based on distinct environmental cues [251]. Engineered probiotics may contain quorum sensing logic gates as part of their design, which allows them to release gelatinase or MMPs under high bacterial densities to assist biofilm dispersion and matrix remodeling processes [210, 252, 253]. Research groups have created ROS- and pH-sensitive promoters which activate the expression of cytokines together with antimicrobials specifically inside inflamed or acidic wound conditions [254–259]. The quorum sensing logic gates enable therapeutic production by probiotics only during situations where their population density reaches levels required for medical intervention. CRISPR-Cas devices linked to nutritional stress serve as self-destroying safety systems to stop unwarranted bacterial expansions [210, 260]. A study has designed two CRISPR-based kill switches for probiotic *E. coli* Nissle 1917 by adding temperature and chemical response elements to promote biocontainment strategies [260]. The research group implemented three biosafety strategies, which incorporated functional redundancy with SOS response control and antibiotic-free plasmid retention to achieve selective engineered strain elimination both inside and afterward the murine intestine. These genetic control systems help safety by reducing unintended gene expression in addition to enhancing effective performance in shifting wound tissue conditions. Such technology permits therapies that respond individually to wound conditions while being beneficial for different wound settings.

Although it has such incredible versatility, the use of CRISPR-Cas9 systems in engineered probiotics has a few limitations and biosafety issues that should be discussed with regard to its clinical translation [261–265]. The major risks would be the development of off-target effects, in which case undesired genomic areas could be edited, causing unforeseen gene perturbations or mutations. Also, Cas9 proteins, and in particular those of *Streptococcus pyogenes* may trigger the host immune system, which presents concerns in relation to inflammation or immunogenicity. Besides, the delivery of CRISPR components into target locations in wound sites or mucosal settings is still problematic as it degrades, inadequately enters the cells, or has leaky expressions. There are also longer-term ecological and genetic safety challenges to the horizontal transfer of CRISPR components and their durable persistence, that is, of concern mainly in complex microbiome environments [266]. These limitations demonstrate the importance of such potent containment mechanisms as kill switches. They also emphasize the great significance of testing medicines adequately in laboratory models to ensure that researchers are certain of accuracy and safety and also ensure that they comply with regulations. Moreover, much complementary molecular and biochemical efforts have been pursued to purposefully augment safety, specificity, and control as scientists progress CRISPR-based technologies to the clinic. To enhance the safety of the CRISPR-Cas system further, especially when applied in therapy, some other strategies may be established [261–265]. (i) Off-targets, which affect the ability of the CRISPR tools to cut incorrect DNA locations, are also a significant threat to genome instability and carcinogenesis. To tackle this, scientists have developed high-fidelity variations of Cas9, e.g. SpCas9-HF1, eSpCas9, and HypaCas9, an approach that decreases nonspecific binding. The others are dual-guide RNA (gRNA) nickase systems and enzyme redesign to increase target specificity. (ii) Genome instability from double-strand breaks (DSBs), traditionally induced by Cas9, can lead to large deletions or chromosomal rearrangements. This Risk can be alleviated by the DSB-free editors, such as base editors (BEs) and prime editors (PEs), and by selecting Cas enzymes with milder activity profiles such as Cas12f and CasMINI. (iii) There is also the issue of immunogenicity and cytotoxicity because the bacterial Cas proteins might lead to the activation of the immune system in the hosts. Chemical modification, such as PEGylation and transient expression systems (based on either mRNA or RNPs) can avoid immune detection. Also, other Cas proteins of less immunogenic species of bacteria are being studied. (iv) Addition of an outside DNA, typically caused by viral inoculation, exposes more possibilities of insertional mutagenesis. DNA integration is a risk, and its use can be avoided with safer delivery systems that include lipid nanoparticles (LNPs) and exosome-based systems. (v) Spatial and temporal regulation of CRISPR is getting more and more significance to prevent unprogrammed editing in the long term. This has resulted in the production of switchable Cas systems that respond to small molecules or light, or pH either through activation or deactivation of editing functions as required by the clinician. As a combination, these complex control systems increase biosafety and specificity of the treatment with CRISPR-based interventions. They are important elements in transforming the CRISPR tools beyond proof-of-concept to clinical practice.

Despite all these safety issues, it is important to note that the field of synthetic biology has made rapid strides to overcome such issues through precision engineering. Rather than being considered purely as limitations, these risks have spurred the design of more and more sophisticated biocontainment and specificity-enhancing approaches such as high-fidelity Cas variants, non-DSB-based editors, switchable Cas, and smart delivery vectors that help advance the safety and clinical feasibility of engineered probiotics using the CRISPR approach [261–265]. Moreover, these advances enable the potential for a new generation of therapeutics which are capable of dynamic responses to wound environments, combinatorial biologics with spatiotemporal control, and even incorporation of diagnostic functionality as part of a biochassis of living microbes. Such a paradigm shift in wound care (from static drugs toward programmable, self-regulating biotherapeutics) could have an impact on the treatment of chronic and infected wounds, particularly for wounds that are resistant to conventional treatment interventions. Therefore, while biosafety remains a crucial pillar of responsible development, it should not overshadow the extraordinary translational promise of these tools. Future progress is going to depend not just on regulatory foresight and strict safety frameworks, but also on further innovation of smart control circuits, delivery platforms and risk mitigation technologies [267, 268]. In this balance lies the way forward: Responsible harnessing of the power of probiotics engineered with the capabilities of the CRISPR to meet the unmet needs in regenerative medicine and infectious disease management.

##### Genetically engineered outer membrane vesicles

Whole-cell probiotics represent one form of probiotics, but engineered outer MVs (OMVs) function as cell-free programmable derivatives. The MVs carry bioactive metabolites and signaling molecules to modulate host immunity. OMVs derived from engineered probiotics may be designed to be loaded with IL-10 or pep2 anti-TNFα peptides or healing peptides such as thymosin β4 or angiogenin [31, 269–272]. These vesicles show better tissue penetration abilities for more efficient delivery as nanocarriers to wound dressings where they enhance wound healing through their immunological activity.

Engineered OMVs have plenty of therapeutic potential but have encountered various obstacles that have to be overcome before making it to clinical translation. Among them is their natural immunogenicity, especially since OMVs may be produced by Gram-negative bacteria that contain lipopolysaccharide (LPS) material components, which may cause accidental inflammation. Moreover, performing large-scale and reproducible production of OMVs with an identical amount of cargo loading is technically difficult since OMV composition may change depending on the conditions of culture and bacterial strain. Considerations have not been made on stability during storage practices as well as on the kinetics of controlled release at physiological conditions. Moreover, a risk of horizontal gene transfer or an accidental packaging of harmful bacterial constituents will have to be properly considered. Such limitations, along with limited purification procedures and preclinical safety testing before use in wound healing therapies, support the necessity of uniformity in the purification procedures and specific, strict preclinical testing of safety.

Scientists can develop advanced wound therapeutic designs through CRISPR precision edits and complex gene circuit designs which start from basic probiotic structures. The living therapeutic agents have two key functions since they detect their surroundings and perform targeted actions to fight infections and accelerate tissue renewal and shape immune cell behaviors, all while staying within the wound tissue. Genetically engineered probiotics show potential as an alternative therapeutic approach for skin wounds and may become practical in the future for chronic and infected as well as immunocompromised situations.

#### Surface engineering: tailoring probiotics for skin wound microenvironments

**Figure 5:**
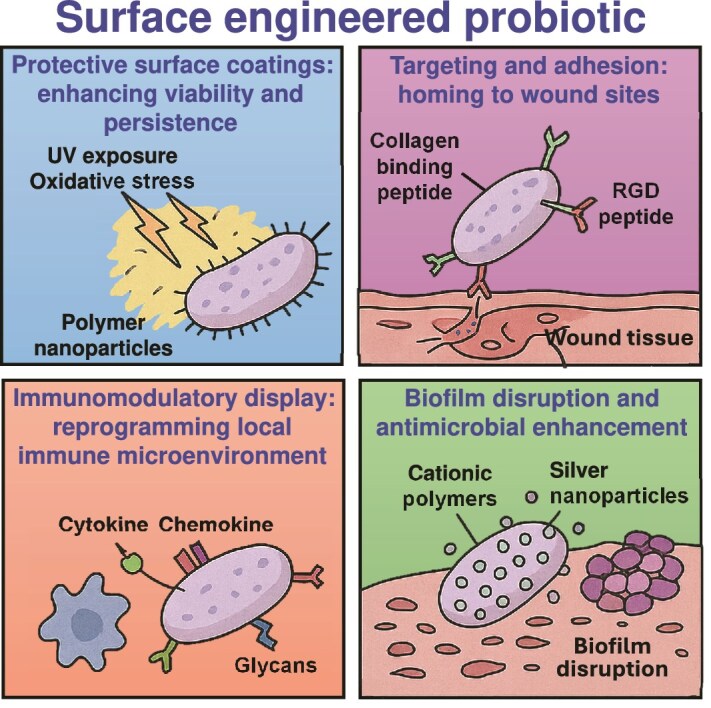
Strategies in surface engineering of probiotics for wound microenvironment adaptation. The schematic divides four major methods of functionalizing probiotic surface in wound treatment applications as: (i) surface protective coatings, using cell-protective polymer nanoparticle layers that protect the probiotic against UV or oxidative stress; (ii) targeting and adhesion using peptides like collagen-binding or RGD motifs for site-specific retention; (iii) immunomodulatory display through the surface expression of cytokines, chemokines, or glycan structures to modulate host immunity; (iv) biofilm disruption and antimicrobial enhancement via cationic or silver-based surface materials that disrupt pathogen biofilms and reduce microbial burden

Genetic engineering gives probiotics new capabilities, yet surface engineering enhances their interactions with their host environment through physical, chemical, and biological modifications [225, 273]. Skin wound healing environments demonstrate hostile conditions which include low oxygen tension combined with low pH and high oxidative stress in addition to pathogenic biofilms and inflammatory debris and fluid and nutrient variations [274]. To overcome these, researchers can develop surface-engineered probiotics to provide better adhesion and protection, along with heightened responsiveness and bioactivity, which establishes an optimal condition between therapeutic probiotics and injured skin tissue. Figure 5 exemplifies four of the exemplary strategies on the surface engineering of probiotics aimed at improving their survival in a complex wound microenvironment, considering targeting precision, immune interaction, and antimicrobial activity.

##### Protective surface coatings: enhancing viability and persistence

The susceptibility of engineered probiotics to rapid loss of function occurs when they encounter open wounds because of both UV exposure and oxidative stress and immune recognition [274, 275]. To overcome this, protective polymer coatings may be applied to their surfaces. The retention of moisture by alginate, chitosan or hyaluronic acid hydrogels which resemble the ECM, allows for enhanced epithelial migration [276, 277]. The surface layers of polydopamine or polyethylene glycol (PEG) serve as stealth factors to protect the bacteria and extend their stay on wound surfaces by limiting immune reactions [278, 279]. For instance, probiotics may be designed to be encapsulated in hyaluronic acid-chitosan nanoparticles to help maintained cell viability on diabetic wounds [31, 280, 281]. The coatings can incorporate stimulus-specific release mechanisms which allow probiotics and therapeutic agents to deliver their compounds exclusively after pH changes or the action of enzymes thus increasing precision while minimizing health risks in the body.

##### Targeting and adhesion: homing to wound sites

Topical probiotics experience a major limitation through their weak retention ability at wound sites because of exudative conditions or wound irregularities. Surface engineering helps improve the adhesive properties between tissue surfaces. The combination of collagen-binding peptides with membrane proteins from probiotics enhances wound attachment since these peptides achieve very tight binding to collagen networks found in dermal wounds [243, 282]. The presence of fibrin-targeting motifs makes probiotics anchor onto blood clot matrices surrounding wound edges. The surface design of arginylglycylaspartic acid (RGD) peptides on probiotics through engineering methods improves their binding abilities to keratinocytes and fibroblasts which contain integrins [225, 283, 284]. Better colonization, together with increased VEGF delivery, can help angiogenesis while promoting granulation tissue formation in the wound healing model.

##### Immunomodulatory display: reprogramming the local immune microenvironment

The designed engineered probiotic surface simultaneously helps wounds transition from chronic inflammation into healing resolution while performing its targeting and stealth functions. Probiotics can be engineered through two methods: (i) by fusing outer membrane proteins with anti-inflammatory cytokines, including IL-10 and TGF-β, or (ii) through glycan motif surface presentation using sialic acid-mimics for reduced innate immune response. The engineering of probiotics allows scientists to add mannose-rich glycan expression through which they capture DCs for regulatory T cell induction leading to faster wound healing in compromised immune settings [285–287]. Additionally, bio-orthogonal click chemistry enables the integration of therapeutic ligands (including AMPs along with anti-TNFα scFvs) onto probiotic surfaces through a process that avoids harming the cells. Click chemistry is a group of very specific, rapid and biocompatible chemical ligation reactions. Bio-orthogonal click chemistry, specifically, encompasses reactions that are not dependent upon native biological processes, that can thus be used to modify the surface of living cells or extracellular vesicles in a manner undisturbing to their normal behavior. This approach allows selective functionalization such as labeling EVs with integrin-specific cyclic RGDyK peptides via cycloaddition of azides with alkynes, to deliver therapeutic reagents such as curcumin to sites of ischemic brain injury in a model system [288]. These kinds of modifications enable delivery to specific locations and maintain the viability as well as activity of the engineered biological vehicle.

##### Biofilm disruption and antimicrobial enhancement

Surface engineering is also applied to give probiotics antibiofilm and antimicrobial properties, particularly important in infected or necrotic wounds. Cationic polymers like ε-polylysine can be used for surface coatings that break pathogenic biofilms by causing membrane depolarization [289–293]. When probiotic surfaces receive light, the dual action of bacterial killing and immune activation is enabled through the attachment design of silver nanoparticles or photothermal agents [294–297]. The integration design of quorum-sensing inhibiting molecules onto surfaces leads to virulence factor suppression in neighboring pathogenic bacteria [298, 299]. Bacteria employ a communication system known as quorum sensing in order to regulate group behaviors, e.g. to produce toxins or assemble biofilm on the basis of population density, by secretion and detection of signals. This symbiosis coordination can be interrupted by preventing quorum sensing thus limiting pathogenicity and consequent complications due to infection. The photothermal nanomaterials may be designed to be integrated onto probiotic surfaces. Upon exposure to near-infrared (NIR) laser activation, the materials create localized heat to destroy MRSA biofilms yet preserve the functioning capacity of probiotic growth factors after activation [300, 301].

Surface-engineered probiotics provide skin wound care with a functional platform that can be readily modified. With surface engineering approaches, probiotics acquire adaptive and responsive qualities through processes that cloak them to extend survival and bond them to wound matrices and adjust their immune interactions. Future research aims to merge genetic engineering with surface manipulation techniques for developing dual-responsive systems wherein probiotic cores run decision processes, yet surface components deliver medicines and interact with wound environments.

#### Nanomaterial-assisted engineering: synergistic probiotic-nanomaterial systems for skin wound healing

**Figure 6:**
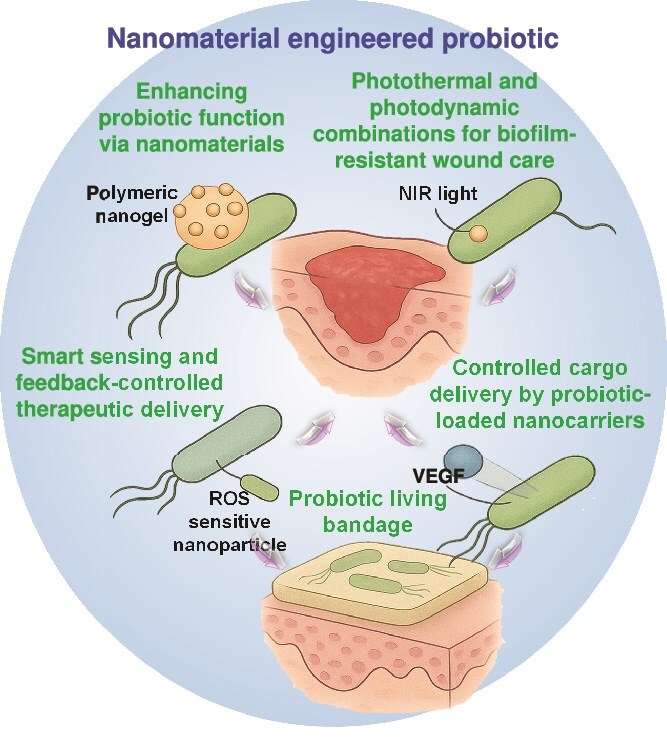
Strategies of nanomaterial-assisted probiotic engineering for advanced wound healing applications. Such schematic is a broad overview of key strategies in NAE of probiotics, including: (i) enhancing probiotic survival and tissue interaction through polymeric nanogels; (ii) achieving photothermal or photodynamic bacterial eradication under NIR light; (iii) integrating ROS-sensitive particles for feedback-controlled therapeutic delivery; (iv) loading nanocarriers onto probiotics for targeted drug such as siRNA or VEGF; (v) incorporating engineered probiotics into smart hydrogel-based living bandages capable of responsive release, inflammation control, and regeneration promotion. These bio-nano hybrid systems combined allow spatiotemporal wound control, pathology-adaptive wound therapies. *NIR* near infrared

Nanomaterial-assisted engineering (NAE) functions as a groundbreaking trend in synthetic microbiology that combines nanomaterials with modern biology [212, 302]. NAE methods combine functional nanomaterials with engineered probiotics to provide dynamic cell adaptability combined with programmable nanotechnological features. The union of these systems produces advanced localized therapeutic approaches that are much more capable than each system could accomplish independently. The platform unites ‘living nano-biohybrids’ that react to wound microenvironment signals and distribute therapeutic substances as well as defend against microbial attacks and initiate tissue healing functions. Figure 6 depicts the major tactics of NAE of probiotics on how such a strategy is integrated into programmable, responsive, and multifunctional wound healing platforms.

##### Enhancing probiotic function via nanomaterials

The healing process needs antibacterial action alongside anti-inflammatory effects together with regenerative properties. Different nanomaterials, including gold nanoparticles (AuNPs) and carbon-based structures together with polymeric nanogels, show promise in improving probiotic tissue attachment for wound treatments by their application to either probiotic surfaces or complete probiotic encapsulation [303, 304]. The protection mechanisms of these materials keep probiotics safe against oxidative damage while also defending them from immune system defense mechanisms. The result is extended survival time. They also can enable external control of probiotic function via stimuli-responsive nanomaterials (e.g. light, pH, ROS) [77, 305]. Probiotics can be engineered to secrete VEGF production, which they encapsulated within pH-sensitive polydopamine-coated nanogels able to protect the probiotics from immune cells thus triggering their controlled release in acidic chronic wound sites for tissue colonization and angiogenesis stimulation [306].

##### Photothermal and photodynamic combinations for biofilm-resistant wound care

The usage of nanomaterials that possess photothermal (PTT) and photodynamic (PDT) properties enables probiotic systems to use on-command disinfection functions for wounds [307, 308]. Graphene oxide (GO) and gold nanorods and copper sulfide nanoparticles receive heat energy from NIR illumination, which enables their ability to kill pathogens and dissolve biofilms [301]. The addition of porphyrin to nanoparticles enables ROS production during specific wavelength irradiation to achieve antibiotic-free bacterial killing [309, 310]. Probiotics can be designed to combine with gold nanoshells placed within a hydrogel wound dressing. When NIR light activates the probiotic-nano hybrid structure, it triggers immediate photothermal destruction of *P. aeruginosa* biofilms [26, 311, 312]. Therefore, this hydrogel wound dressing may design to release IL-10 by the underlying probiotics, suppressing local inflammation and accelerated wound closure compared to conventional antibiotic dressings [313, 314]. The ‘photothermally activated probiotic dressing’ shows how nanomaterials can enhance probiotic protection along with controllers of treatment duration.

##### Smart sensing and feedback-controlled therapeutic delivery

Real-time sensing and feedback loops integrated into modern NAE platforms drive precision wound care processes [315, 316]. ROS-sensitive nanocarriers may be designed for triggering the release of probiotics or cytokines only in inflamed wound zones [317]. Glucose-sensitive hydrogels can be designed for the hyperglycemia-triggered probiotic-releasing diabetic wound dressings. Probiotics attached can be combined with iron oxide nanoparticles, enabling magnetic steering toward complex or deeply located wound areas [318, 319]. Probiotics may be designed to be with MnO₂ nanoparticles that react with hydrogen peroxide in infected wounds, generating oxygen bubbles that enhanced probiotic dispersion and survival [26, 320, 321]. Simultaneously, probiotics can be designed for the production of hyaluronidase and AMPs to break down biofilm and enhance granulation tissue formation [322].

##### Controlled cargo delivery by probiotics-loaded nanocarriers

The therapeutic strategy employs nanomaterials as transport agents that probiotics deliver through their bodies directly to wounds. The delivery system involves using nanoparticles made from lipid-based carriers, which contain siRNAs and anti-inflammatory drugs and angiogenic factors attached to probiotic membranes. A compound of probiotics combined with polymeric nanoparticles functions as motile carriers which improves nanoparticles’ penetration capabilities into deep tissues. The surface of probiotics can be designed with siTNF-a-containing liposomes to specifically address inflammatory processes within chronic wounds. The wound-attaching probiotic agent allows the liposomes to be released based upon cytokine levels, which reduced TNF-α and enhanced skin cell regeneration [227, 323].

##### Next-generation concepts: probiotic living bandages

Researchers can combine nanomaterials and probiotics into modular wound dressings through two approaches such as smart hydrogels that contain probiotic-nanoparticle hybrids or 3D bioprinted patches enabling precise placement of probiotics and nanomaterials. Films containing self-healing properties enable the gradual release of probiotics and nanodrugs upon mechanical forces or microtears in the material structure [324, 325]. Active dressings have the ability to detect, react and heal like the intelligent mechanisms found in biological tissues [326, 327]. Some versions are designed to fluoresce under infection, acting as diagnostic indicators for early intervention.

The implementation of nanomaterial engineering enables probiotics to develop into responsive biotherapeutics that are safe and exhibit multiple therapeutic functions for wound healing in the skin. The combination of nanotechnology precision and adaptive metabolism of probiotics enables us to design advanced wound treatments that respond to pathology while delivering precise actions with enhanced regenerative signals that exceed individual technologies’ capabilities. Such systems function as more than simple treatments while simultaneously acting as responsive tissue that connects synthetic biological techniques with human body dynamics.

### Engineered probiotic applications in skin wound healing

The principles of genetic and surface engineering led to recent developments of engineered probiotic platforms intended for skin wound healing improvement. Engineered probiotics now demonstrate an active role in regulating the immune microenvironment and delivering therapeutic agents alongside their ability to adhere to wounds. The therapeutic effects of engineered probiotics with associated delivery enhancement strategies for skin wound healing are described systematically in Tables 5 and 6. The engineering methods for developing probiotics consist of genetic modification (Table 5) and material-assisted encapsulation and membrane vesicle extraction techniques (Table 6). This unified research presents insights about the developing field that uses engineered probiotics as an alternative therapeutic medicine for regenerative medicine purposes.

**Table 5:** Genetically engineered probiotics for wound healing

Probiotic	Engineering strategy	Application form	Dosage	Model	Observed effect	Reference
*L. reuteri*	Genetically engineered *L. reuteri* expressing CXCL12 (ILP100)	Topical application of live engineered bacteria	Optimal dose (2 × 10^7^ CFU/wound) daily	Mouse skin wound model, diabetic and ischemic mouse wound model, human skin ex vivo wound model	Increased TGF-β and M2 polarization, accelerated wound closure, improved revascularization	Vågesjö and Phillipson et al., 2018 [45]
	Genetically engineered *L. reuteri* expressing CXCL12 (ILP100)	Topical application of live engineered bacteria	ILP100: 7 × 10^9^ CFU/wound (100 μL); Wild-type: 2.5 × 10^9^ CFU/wound (500 μL)	Minipig wound model	Induced macrophage recruitment and increased TGF-β production, accelerated granulation tissue formation and re-epithelialization	Öhnstedt and Phillipson et al., 2022 [46]
	Genetically engineered *L. reuteri* expressing CXCL12 (ILP100)	Topical application of live engineered bacteria	1 × 10^7^ CFU/cm^2^ (clinical dose) on agar	*In vitro* MDR pathogen inhibition in simulated wound fluid	Local pathogen suppression, enhanced healing microenvironment	Lofton-Tomenius and Phillipson et al., 2025 [47]
	Genetically engineered *Lactobacillus reuteri* expressing CXCL12 (ILP100)	Topical application of live engineered bacteria	Single dose or Multidose (5 × 10^4^, 5 × 10^7^, 1 × 10^9^ CFU/cm^2^)	Human Phase 1 trial for skin wound healing	Faster closure, safe profile	Öhnstedt and Phillipson et al., 2023 [43]
	Genetically engineered *L. reuteri* expressing CXCL12 (ILP100)	Topical application of live engineered bacteria	High dose (1 × 10^9^ CFU/cm^2^), Low dose (5 × 10^7^ CFU/cm^2^)	Human diabetic foot Ulcer patients	Terminated due to patient recruitment issues	NCT05608187-RCT phase 2a
*L. cremoris*	Genetically engineered *L. cremoris* expressing FGF-2, IL-4, and CSF-1 (AUP1602-C)	Topical liquid suspension of *L. cremoris* AUP1602-C	2.5 × 10^7^ CFU/cm^2^ daily for ^7^ days in mice; 2.5 × 10^6^ CFU/cm^2^ or 2.5 × 10^8^ CFU/cm^2^daily for 7 days in minipig	Mouse diabetic (db/db) skin wound model and minipig wound model	Accelerated wound closure; enhanced re-epithelialization and collagen deposition	Kurkipuroet al., 2022 [48]
	Genetically engineered *L. cremoris* expressing FGF-2, IL-4, and CSF-1 (AUP1602-C)	Topical liquid suspension of *L. cremoris* AUP1602-C	2.5 × 10^5^ CFU/cm^2^ (single dose); 2.5 × 10^6^ to 2.5 × 10^8^ CFU/cm^2^ (3×/week for 6 weeks)	Human diabetic foot Ulcer patients	Well-tolerated; dose-dependent efficacy; complete healing in 83% at highest dose; improved QoL and granulation tissue formation	Schindler et al., 2024 [44]

*CSF* colony-stimulating factor-1, *CFU* colony-forming unit, *FGF* fibroblast growth factor, *IL* interleukin, *MDR* multidrug resistance, *M2* anti-inflammatory M2 macrophage, *QoL* quality of life.

**Table 6:** Material-assisted delivery systems for probiotics in wound healing

Delivery strategy	Common probiotics used	Engineering strategies	Typical application forms	Typical dosages	Major observed effects	References
Hydrogel	*L. plantarum, Lactobacillus rhamnosus, Lactobacillus reuteri*	Schiff-base crosslinking hydrogel (ProGel), honey bioactive gel, dual-layer hydrogel dressing (guar gum/PVA), hyaluronate-based self-healing hydrogel (HPF), GelMA hydrogel, pH/ROS dual-responsive SA-SPBA hydrogel, alginate microspheres in GelNBSH-L bioprintable hydrogel, microspheres in hyaluronic acid hydrogel, chitosan/hyaluronan hydrogel, chitosan nanogel	Injectable hydrogel, topical gelation, bioprintable hydrogel	~10^7^–10^9^ CFU/ml; or probiotic lysates 1% (w/w)	↑ Antibacterial, wound healing, collagen deposition, re-epithelialization, angiogenesis, lactic acid secretion, regeneration; ↓ Biofilm, quorum sensing, inflammation; Modulated wound microbiota, protected probiotic viability	[118, 134, 154, 157, 168–173]
Nanofiber/Scaffold	*L. plantarum, Lactobacillus acidophilus, L. rhamnosus, Lactobacillus casei*	LAB-ZnO NPs hybrid, multi-layered electrospun PU/gelatin/PU scaffold, electrospun nanofibers, SF/SA scaffold, macrogel/microgel encapsulation, chitosan scaffold	Topical nanofiber dressing, topical multilayer scaffold, implanted scaffold	~10^8^ CFU/ml, lysate from probiotics (~10^9^ CFU/ml)	↑ Antibacterial, wound healing, fibroblast activation, angiogenesis, antioxidant, M2 polarization; ↓ Biofilm, cytotoxicity, infection, TNF-α	[122, 174–178]
Oleogel	*L. rhamnosus, L. acidophilus, Lactobacillus fermentum, L. casei, L. reuteri*	Direct incorporation into oleogel base	Topical gel or ointment	1 × 10^9^ CFU/ml viable cells	↑ Wound closure, re-epithelialization, collagen deposition, neovascularization; ↓ MPO activity, inflammation	[127, 179]
Membrane vesicles	*L. plantarum, L. casei, L. reuteri*	MV extracted from probiotics and hydrogel embedding	Topical hydrogel with MVs (hydroxyethyl cellulose)	MVs: ~10^11^–10^12^ particles/ml or 200 μg/wound	↑ Wound healing, re-epithelialization, vascularization, M2 polarization, IL-10; ↓ Neutrophil infiltration, TNF-α	[120, 121, 180]
Others	*L. plantarum, L. reuteri, L. casei*	Curcumin-loaded solid LNPs, acid-responsive nanoparticles, NO donors forming bacteriophage-like microparticles, kaempferol forming microparticles, BNC-HA nanocomposites	Topical biodegradable sponge, topical application, spray thin film	~1.04 × 10^8^ CFU probiotics and 256 μg curcumin/sponge; 2 mg/ml probiotic extract+0.3 mg/ml NO donor	↑Antibacterial, MRSA killing, wound healing, angiogenesis (VEGF, TGF-β), antioxidant (catalase, GSH, SOD), M2 polarization, NO-mediated immune modulation; ↓ ROS, inflammation (TNF-α, MMP-9)	[136, 148, 149, 181, 182]

↑: increase; ↓: decrease; *BNC-HA* bacterial nanocellulose–hyaluronic acid nanocomposite, *GelMA* gelatin methacryloyl, *GelNBSH-L* GelNB-GelSH hydrogel, *GSH* glutathione, *HPF* human pulmonary fibroblasts, *IL* interleukin, *LAB* lactic acid bacteria, *M2* anti-inflammatory M2 macrophage, *MVs* membrane vesicles, *MMP* matrix metalloproteinase, *MPO* myeloperoxidase, *MRSA* methicillin-resistance *S. aureus*; *NPs* nanoparticles, *SF/SA* silk fibroin/sodium alginate, *SPBA* 4-Formylphenylboronic acid-modified succinylated polyethyleneimine, *SOD* superoxide dismutase

#### Genetic modification strategies

Wound-site production of therapeutic molecules through engineered probiotics provides dual regenerative abilities together with immunomodulatory properties. ILP100-Topical represents a pioneering approach as a CXCL12-producing genetically engineered *L. reuteri* platform that shows potential as a representative platform. Phillipson’s team developed ILP100-Topical based on their experimental outcomes and subsequently advanced it to phase II clinical trials. Phillipson *et al.* demonstrated that the topical application of *L. reuteri* expressing CXCL12 accelerated wound healing in mice by enhancing dermal cell proliferation and promoting macrophage polarization toward a reparative phenotype in 2018 [45]. This research team confirmed the improved tissue granulation function and skin re-epithelization of ILP100-Topical by testing it on minipigs in 2022 [46]. Their group in 2025 demonstrated that antimicrobial properties functioned effectively in ILP100 against highly MDR wound pathogens derived from war injuries, surpassing conventional antibiotics in laboratory tests, thus showing additional anti-infective capabilities of this probiotic drug candidate [47]. The SITU-SAFE clinical trial in 2023 demonstrated both acceptable safety and promising initial effects of ILP100-Topical on wound healing and vascular network development when used in healthy participants [43]. This trial took place in 2023 under double-blind, randomized and placebo-controlled conditions. Nevertheless, although the trial had a randomized, controlled, placebo, and double-blinded design, some methodological limitations are to be considered. It is important to mention that the research was those that may be limited because it was done in only one center, and the sample size used was relatively small. Compositions of clinical investigator teams of the study changed, especially in the MAD cohort 2, which might have caused inconsistencies in procedures, especially wound care and evaluation. Also, the sample population in the study was made up solely of healthy non-obese people who were under the age of 45, which is not reflective of the average patient with a chronic wound, who are frequently diabetic, have vascular insufficiency, or are immunocompromised. This may open up a translational gap between the controlled clinical environment and the actual patients in the real world, where wound healing issues are complicated. Moreover, it can be noted that despite the observed follow-up period of adverse events of up to 13 months, genetically engineered probiotics as a source of human therapy are still a rather new modality. Considering the possible risks of induced, long-term, or delayed immune reactions, a longer follow-up on safety might be desirable to obtain a fuller coverage of the chronic effects or the latent risks. This is especially relevant in genetically modified organisms, ones that operate *in vivo* and over time interact with tissue environments. It would also be noted that the Phase 2a trial (NCT05608187) to specifically assess ILP100-Topical in a clinically more relevant patient population, such as patients with DFU, is currently temporarily stopped as they reportedly had difficulties recruiting participants. Although this kind of logistical restriction is not uncommon in early clinical development, it still restricts the imminent availability of confirmatory data of efficacy in populations of individuals with impaired healing. In addition, the exclusion criteria of the trial, i.e. the lack of participants with infected or heavily exuding patients whose wound duration exceeded 2 years, heavily exuding ulcers, severe renal impairment (eGFR <30 ml/min/1.73m^2^), hemoglobin levels <100 g/L, use of corticosteroids or immunosuppressants, active Charcot foot, and recent modifications in diabetes drugs, and current smokers, can also limit the generalizability of the results. In order to improve the external validity in further research, one should extend the inclusion criteria, stratify randomization, and recruit internationally or across many centers. Real-world evidence (RWE) supplementation and adaptive designs of trials might also involve a better representation of the safety and efficacy concerns, particularly in the populations that have multifaceted issues with wound healing. The therapeutic potential of ILP100-Topical in complex wound situations may be promising, but early pending the availability of additional outcomes. Moreover, the most recent single-cell transcriptome experiments have found fibroblast-derived migrasomes as a provider of CXCL12 delivery in the wound bed, and this migrasome helps serve as a local version of the carrier of epithelial proliferation and neovascularization, reinforcing the biological premise of the CXCL12-facilitated wound healing [328].

Additionally, AUP1602-C is a next-generation, multi-target gene therapy in the treatment of chronic wounds [44]. AUP1602-C is a transgenically modified variant of *L. cremoris* that expresses three synergistic human therapy productive proteins locally and secreted to the site of the wound, i.e. fibroblast growth factor-2 (FGF-2), IL-4, and colony-stimulating factor-1 (CSF-1). All these factors have a synergistic effect of modulating immune microenvironment, polarizing macrophages toward regenerative M2, stimulating fibroblast proliferation and angiogenesis, and favoring epithelialization. Through application of localized combinatorial regenerative stimulus, the AUP1602-C acts like millions of biological micro-factories that reactivate stuck healing mechanisms in chronic DFUs. Kurkipuro *et al.* showed that AUP1602-C pre-clinically significantly accelerated wound closure relative to control groups, promoted re-epithelialization, and promoted the formation of granulation tissue and conformed to its suggested biological mechanism of action with genetically diabetic (db/db) mice in 2022 [48]. Schindler *et al.* conducted a first-in-human (FIH), open-label Phase I dose-escalating study (NCT04281992) in 2024, showing that AUP1602-C is safe, well tolerated, and has dose-dependent efficacy in patients with non-healing DFUs [44]. The complete wound closure and absence of recurrence were achieved in 83% of patients in the highest dose group (2.5 × 10^8^ CFU/cm^2^). There were no serious adverse events associated with the treatment and the most common treatment-emergent adverse event was skin maceration of a mild and reversible type. Although preliminary results of a Phase I clinical trial of AUP1602-C point to potential benefits in terms of safety and efficacy in the treatment of nhDFUs, some limitations deriving from the study design make the results less interpretable and generalizable. The fact that the study employed an open-label, nonrandomized, and uncontrolled design, although well adopted and expected in FIH trials, comes with the possibility of bias or a source of bias, as in selection, performance bias, and assessment bias, which may influence the observed treatment effect. It is difficult to determine the therapeutic effect of AUP1602-C without a placebo group or active comparator group, as the variation in wound healing is natural, and as patients can react differently to standard care. Moreover, the study was performed at one clinical site with a small number of participants (*n* = 16) split into four dosing cohorts that gradually increased. Although such a sample size is suitable to carry out preliminary safety, it in itself limits the statistical strength and outside applicability of the resultant efficacy outcomes. Baseline difference in other characteristics, including off-loading compliance, ulcer duration, and glycemic control, may also confound cohort-level comparison. The study will allow us to view the results of long-term efficacy 12 months after the start of treatment, which allows useful insight about the medium-term survivability of the treatment, but the issue of long-term safety when genetically engineered live probiotics are used to treat chronic wounds still remains an aspect awaiting more observation. Although no immunogenic or systemic adverse events were observed, the future challenges potentially identified, such as delayed interactions between a host and a microbiota, horizontal gene transfer, or shedding of therapies to the environment, although theoretical, stress the necessity of long-term pharmacovigilance due to this being a relatively new type of therapy. Finally, although a claimed 83% wound closure at the highest dose level, and no recurrence within a 12-month follow-up is encouraging, these findings are to be treated with caution, as they occur without a matched control group, especially considering the high recurrence rate of DFUs inherently. Such a more rigorous test, ideally through a randomized, controlled, multi-center trial, will be necessary to establish efficacy and safety in different, broader and more heterogeneous patient populations. Encouragingly, a Phase II study is being conducted in Europe to answer such sequential clinical questions. Similar to ILP100-Topical, AUP1602-C exemplifies the growing promise of live biotherapeutic products (LBPs) in regenerative medicine. But its triple-cytokine engineering exhibit points to broader immunomodulatory and tissue regenerative properties. Such a multi-factorial platform exemplifies how the potential of engineering probiotics may be utilized to address the complex wound pathophysiology by hitting multiple healing pathways at the same time, which has the potential to contribute to chronic wound treatment.

While the early results of ILP100-Topical and AUP1602-C are encouraging, the current limitations in trial design and the characteristics of the population are considerably limiting their translational potential [43, 44]. The overly selective inclusion criteria (e.g. non-obese, healthy adults <45 years old in ILP100-Topical; and limited sample size without a control group in AUP1602-C) do not represent the complex co-morbidity commonly seen in chronic wound patients. This discrepancy leads to concerns about efficacy that is seen in ideal conditions, not having the potential to spread to the larger clinical population, especially those with diabetes, vascular insufficiency, and/or immune dysregulation. Moreover, the single-center nature of these trials leaves geographic and procedural biases and the lack of randomization (or comparator groups, especially in AUP1602-C) prevents making causal inferences. These limitations may result in overestimation of efficacy or underreporting of adverse events, which may result in delayed regulatory approval and use in regular pathways of care. In order to overcome these barriers, future clinical development will need to be designed with more translationally relevant trial designs, including: (i) Multi-center recruitment of a diverse demographics and wound etiology. (ii) Randomized, placebo-controlled, double-blind protocols to decrease bias. (iii) More broad inclusion criteria, especially targeting patients with chronic or infected wounds. (iv) Longitudinal safety monitoring to evaluate delayed immune responses or risks of environmental shedding. (v) RWE integration and adaptive trial frameworks to link clinical and practical outcomes. In addition, regulatory pathways for genetically modified live biotherapeutics are also complex [267, 268]. Therefore, early communication with regulators (e.g. EMA, FDA) and the implementation of biosafety monitoring systems (e.g. kill switches, horizontal gene transfer barriers) could help de-risk development. These types of strategies are going to be important in moving engineered probiotics beyond the experimental level to a level where they are routinely going to be used in wound care. These studies confirm ILP100-Topical and AUP1602-C as the innovative first-generation engineered probiotic therapy for wound care that achieves local chemokine/cytokine delivery and immune system control while also providing antibacterial properties. This platform highlights the tremendous clinical potential of synthetic probiotics in regenerative medicine and infectious disease management.

#### Material-assisted encapsulation strategies

Wound environments receive enhanced delivery of bacteria because encapsulation and scaffold technologies help preserve probiotic viability and maintain biological activity. The representative delivery platforms for probiotics involve hydrogel encapsulation systems, nanofiber or scaffold-based delivery, and oleogel-based formulations. (i) Hydrogel encapsulation systems: researchers developed pH and ROS dual-responsive alginate hydrogels crosslinked with SPBA to encapsulate *L. rhamnosus*, which released the probiotics under oxidative stress to enhance bacterial clearance and wound healing processes [168]. Self-healing injectable hydrogels containing *L. rhamnosus* and hyaluronate-adipic dihydrazide/Pluronic F127/fucoidan improved collagen production and reduced inflammation [169]. The infection control capabilities and speed of healing improved when *L. rhamnosus* LGG metabolites were incorporated into photopolymerized GelMA hydrogels [170]. Researchers developed culture-delivery bioprintable hydrogels that incorporated *L. reuteri* in alginate microspheres to achieve antimicrobial effects which maintained >90% inhibition against *S. aureus* and *E. coli* [118]. Infected wounds showed accelerated healing after being treated by living *L. reuteri* located within hydrogel microspheres before their transfer into photocrosslinked HA hydrogels [134]. The incorporation of *L. reuteri* into a metal-phenolic self-assembly (FeTA) protected hydrogel made from carboxylated chitosan/oxidized hyaluronan protected the probiotic bacteria from antibiotic harm [171]. Wound dressings consisting of dual layers containing *L. plantarum* hydrogels with hydrocolloids led to full re-epithelialization of surfaces [172]. (ii) Nanofiber or scaffold-based delivery: the antibacterial and antibiofilm properties of NPs that combine LAB-ZnO and nanofiber scaffolds were achieved through the biosynthesis of *L. plantarum* and *L. acidophilus* [174]. The combined use of *L. rhamnosus* EPS and bioactive glass nanoparticles in electrospun nanofibers produced materials with antibacterial and antioxidant behavior [175]. The release of *L. casei* along with sustained lactic acid production from silk fibroin/sodium alginate scaffolds directed M2 macrophage polarization and speeded up angiogenesis [122]. Bioactive hydrogel dressing systems manufactured through combination of Macrogel/Microgel encapsulation techniques delivered sustained probiotic functionalities [176]. (iii) Oleogel-based formulations: research revealed that applying oleogels comprised of *L. acidophilus*, *L. rhamnosus*, and *Lactobacillus fermentum* enhanced fibroblast proliferation and promoted wound healing in diabetic rats [127]. Engineered probiotic therapeutics receive strong protection and controlled drug release benefits while offering multi-functional effects through material engineering approaches.

#### Membrane vesicle extraction strategies

MVs extracted from probiotics offer a new delivery method that bypasses the need to maintain live bacterial survival. Scientific reports have demonstrated that *Lactobacillus*-derived MVs used in hydrogel compounds enhanced vascularization pathways while decreasing neutrophil migration alongside altering inflammatory response patterns [120, 121, 180]. The integration of MVs derived from *L. plantarum* and *L. casei* into biomimetic hydrogels promoted M2 polarization of macrophages as well as improved tissue reconstruction [120]. The MV-based technology sector stands as an emerging promising field that provides bioactive methods to advance wound healing without requiring living cells.

Engineered probiotic treatments for wound healing continue toward rapid advancement through methods that include genetically reprogrammed organisms and material delivery systems and vesicular systems. Alternative engineered probiotic medicines show great promise for regenerative dermatology and trauma recovery because they unite three essential therapeutic features: immune regulation, antimicrobial effects and tissue reconstruction initiation. Additional research may concentrate on enhancing delivery platforms and enhancing targeting precision while conducting comprehensive clinical studies to achieve the optimal therapeutic effects of engineered probiotics in wound treatment.

### Future perspectives

**Figure 7:**
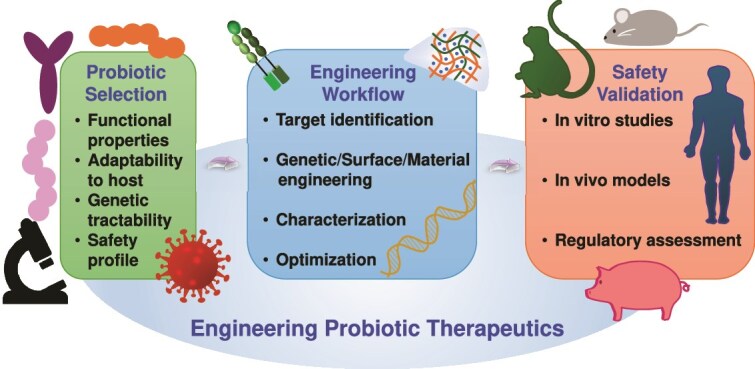
Key framework for engineering probiotic therapeutics. Flowchart of the three key pillars in the process of designing engineered probiotic-based therapies: (i) probiotic selection integrating functional, genetic, and safety features; (ii) engineering pipeline integrating genetic, material and characterization components; (iii) safety-certification comprising preclinical and regulatory experimentation

Probiotic-based skin trauma treatment will advance through the united development of microbiology, materials science, synthetic biology and trauma medicine. Although encouraging data in both murine and minipig studies have been reported, the main challenge to conducting engineered probiotic therapies in human beings is their translation barriers. The first notable challenge is that the human skin microbiome is highly inter-individually variable due to the genetic background of a host, age, previous use of antibiotics, underlying conditions like diabetes, and environmental exposure. Such diversity not only influences the colonization capability and viability in the respective probiotic strains applied, but it also alters the immunological effect of the strains. As an illustration, normative DFU wounds tend to have high concentrations of pathogenic bacteria that compete with therapeutic strains, thus reducing their efficacy and the rate of engraftment. Additionally, host immune reactions to engineered bacteria also present an extra complication to clinical translation of live biotherapeutics. A study printed that it is intended that engineered strains can be used to synthesize therapeutic agents or tune a host response, and that their site of presence might unintentionally lead to an activation of the immune system [329]. In particular, genetically modified strains that express heterologous molecules like cytokines or peptides may react unpredictably with the host immune system and cause it to be not only overstimulated but also lose immune tolerance. These effects may be local inflammation, immune activation, or antibody production that are neutralizing in nature, especially when the administration of strains is in repeated forms over a long period of time. Moreover, ecological imbalance and horizontal gene transfer may aggravate the pathology of immune imbalance or even give way to dysbiosis. Hence, stringent preclinical immunotoxicity studies and longitudinal immune surveillance in human studies are important so as to ensure that the engineered strains would not trigger the development of harmful or unintended responses in a host. To eliminate the above risks, biosafety design should have features like kill switches and immune stealth design in future development. Multiple scientific fields create a convergence which turns into actual knowledge exchanges between microbiology research and bioengineering developments to tackle key wound treatment challenges. Wound healing practices based on passive dressing techniques and antimicrobial interventions have reached a point of limited effectiveness for treating chronic wounds as well as infected trauma sites and wounds in immunocompromised patients. Engineered probiotics provide a therapeutic modality because living organisms can sense conditions and reshape the wound environment actively and respond to their environment adaptively. Figure 7 summarizes this multi-dimensional development process as a flow scheme, which overviews the main modules of probiotic therapeutic engineering—choice of strain, optimization of genetic/material characteristics, as well as safety verification within *in vitro* and *in vivo* environments. Intelligent wound healing platforms incorporating engineered probiotics and smart biomaterials together with real-time biosensing technologies will advance through development. The envisioned system turns probiotics into smart therapeutic agents which perceive inflammatory signals and control their active substance delivery while responding to feedback from the wound environment. This multidirectional therapeutic interaction would be built by materials science through hydrogels and nanofiber meshes along with self-healing matrices that protect probiotic life while modifying their therapeutic effects. Synthetic biology incorporates logic circuits into probiotics, which allows them to act as synthetic immune cells that recognize specific environmental stimuli. Embedded biosensors in the system will monitor pH and ROS levels and cytokines and bacterial counts through which real-time control mechanisms adjust microbial functions and hydrogel mechanics together with drug dosage algorithms. A *smart trauma healing system* will convert traditional wound dressing into an active living platform that links biological functions to medical intervention. Healthcare providers could deploy adaptive therapeutic systems that learn about healing stages so they can control infections during early periods, while helping tissue development during mid-phase, and reducing scar effects during the remolding stage. In this envisioned system, living probiotics would not simply be passive occupants but active participants—sensing local inflammatory cues, dynamically releasing therapeutic payloads such as cytokines, growth factors, or antimicrobials, and self-regulating their behavior based on wound status feedback. Materials science would provide the scaffold for this dynamic interplay, designing hydrogels, nanofiber meshes, or self-healing matrices that not only protect probiotic viability but also amplify and guide their therapeutic actions. Synthetic biology would endow probiotics with sophisticated logic circuits, enabling decision-making capabilities akin to synthetic immune cells that respond selectively to environmental stimuli. Meanwhile, embedded biosensors could monitor parameters such as pH, ROS, cytokine levels, or bacterial burden, feeding data into closed-loop control systems that modulate microbial activity, hydrogel properties, or drug release kinetics in real time. Such a ‘smart trauma healing system’ would transform static wound dressings into dynamic, living interfaces between biology and medicine. Instead of applying repeated antibiotic treatments or surgical debridement reactively, clinicians could deploy responsive therapeutic systems capable of adapting to each stage of healing—controlling infection during early phases, promoting angiogenesis and fibroblast proliferation during mid-phase, and minimizing scarring during remodeling. Engineered probiotics which benefit from genetic or surface modifications exhibit natural immunomodulatory properties that allow fine control of inflammation and wound healing processes thus accelerating the wound repair process without structural damage to the tissue. A transformation in wound therapeutics becomes possible through the integration of clinical trauma management with microbiology along with material engineering and synthetic biology techniques. Human trials of engineered probiotics are likely to drive future developments toward modular wound therapies, which can be adapted to treat distinct wound types and personalize treatment for each patient’s unique microbiome and immune profile. The custom-built smart therapeutic approach will generate active healing rather than just treating wounds to create a novel frontier within regenerative medicine.

Various significant hurdles stand in the way of achieving intelligent engineered probiotic systems for wound healing despite their impressive progress. Paramount among these is biosafety. The strong therapeutic effects of engineered live probiotics generate safety worries because they may spread genetic material to other organisms and freely multiply within the environment [29, 32, 34, 210]. Besides issues on genetic propagation and unregulated duplication, the rising literature shows that there are several limitations, both intrinsic and extrinsic, of probiotics [330]. Although most commonly viewed as safe, some probiotic strains may, under certain conditions, switch from being harmless to becoming opportunistic pathogens when introduced to a new environment (or a new host). Intrinsic risks are related to the likelihood that dangerous metabolites or virulence-related enzymes such as collagenases and hemolysins will be produced, that antibiotic resistance genes will horizontally transfer through a system, and uncontrolled immune events will occur. Certain probiotic species, notably *Lactobacillus*, *Enterococcus* and *Bifidobacterium* have been associated with clinical infections, including bacteremia and endocarditis, and also abscess formation, especially in immunocompromised patients or patients with indwelling medical devices [331, 332]. Overall biofilm formation, which is useful in mucosa colonization and immune training, may also lead to extended persistence, host resistance, and avoidance of host immune response and resistance to antibiotics. As an example, overdeveloped, mislocalized probiotic biofilms have been linked to small intestinal bacterial overgrowth (SIBO) and chronic inflammation in predisposed organisms [330, 333, 334]. The uncertainty of quality control is further increased when there is no standardization of commercially available probiotic products, causing variable viability, strain identity and functional properties between batches available. Bearing in mind these risks, clinical use of live engineered probiotics should be treated cautiously especially among susceptible groups. Such strategies as rigorous biosafety testing, genetic containment methods (e.g. auxotrophy circuits, or kill switches), and host-specific risk stratification should be viewed as obligatory elements of the probiotic system design. Recent research has proved that probiotic biofilm, particularly those of *L. reuteri* or other sources, still have their immunomodulatory and osteogenic capabilities even after pasteurization, effectively resolving the issues related to a live bacterial infection [335–337]. The application of inactivated or pasteurized probiotic biofilms not only prevents the risks of biosafety, like sepsis or uncontrollable bacterial growth, but also maintains the property to induce tissue repolarization in macrophages toward the anti-inflammatory M2 types and resistive tissue recovery, and as such is a potential source of translation to clinical practices in wound healing and bone repairing. The incorporation of auxotrophy circuits along with kill switches and self-limiting designs constitutes essential strategies to address these safety hazards in microbial engineering workflows [31, 338, 339]. As an example, D-alanine auxotrophy is based on the idea that D-alanine forms an important part of bacterial peptidoglycan that is needed in cell wall production. The resulting engineered strains lacking alanine racemase (*alr*) or dadX cannot convert L-alanine to D-alanine and will thus require exogenous D-alanine as their sole source of growth [340, 341]. Since the D-alanine is not generally supplied in the natural tissues, such strains are not able to exist *in vivo* or even in environmental terms without being provided. In comparison to conventional auxotrophies (e.g. D-alanine auxotrophy), Mandell *et al.* have now developed *E. coli* strains whose genetic coding has been rewritten to use the synthetic amino acid (bipA) L-4,4-biphenylalanine in critical proteins. In the absence of bipA, strains cannot survive or propagate, and bipA is not found in the environment or human physiology, thus removing the risk of an environmental escape or horizontal gene transfer [342]. In this mechanism, all of the native TAG stop codons present in the bacterial genome are replaced with TAA meaning that the TAG codon itself is available as a reassignment to bipA. Also, the strain engineered possesses a recoded essential gene (e.g. adk) which should have the bipA inserted at a specific location to allow successful protein functioning. In the absence of sustained external supplies of bipA these important proteins lose their functionality and the cell quickly perishes, thereby offering a strong and evolutionary stable containment strategy. This strategy is sturdier, since the bacteria are completely reliant on a fabricated metabolite which is not present in the identified biome, lowering the danger of escape through environment complementation, or by mutation reversion [343]. Such metabolic dependencies are invertible and adjustable, providing a dynamic source of spatial and temporal control of the microbial viability. Further, in combination with other biosafety measures (e.g. kill switches or environmental sensing circuits), auxotrophy constitutes a fundamental element of effective live biotherapeutic product biocontainment systems. A second layer of biosafety protection is the incorporation of synthetic biocontainment circuits (including genetically encoded kill switches and lysis modules) that induce bacterial self-destruction of the bacteria in the event of escape from the therapeutic niche or encounter with non-permissive environmental conditions [42, 344]. Such kill switches are built based on systems such as toxin-antitoxin systems (e.g. CcdB) in which a deadly gene (toxin) is strongly controlled by the conditions of the environment [42]. Leaving permissive conditions (e.g. the microenvironment of a wound in the host), an environmentally responsive promoter, e.g. the temperature-sensitive PcspA, becomes activated, de-repressing the subsequent toxin expression. As a solution to regression to leaky expression under non-inducible conditions, a basal level of antitoxin that is constitutively expressed is co-expressed to counteract any otherwise accidental toxin expression, and hence stable and non-penalizing maintenance of the co-expression system occurs against this *in vivo*. Also, evolutionarily stable structures, including the systems described by Stirling *et al.* as the essentializer and cryodeath, provide examples of how kill switches can be adjusted through rational design of ribosome-binding sites (RBSs) and promoters to establish a low escape rate (≤1 in 10^5^ over 140 generations) [344]. The designs also highlight the value behind fitness-neutral expression between toxin and antitoxin, where they allow avoiding loss-of-function mutations, commonly occurring when synthetic burdens reduce the viability of a host. These principles have also been extended recently by using CRISPR-based kill switches. *E. coli* Nissle 1917 that contains engineered CRISPR-Cas9 kill switches with one-input (chemical) and two-input (chemical + temperature) control were designed to target multiple chromosomal loci [260]. They combined several parallel solutions like multi-locus targeting, manipulation of the SOS pathway, plasmid retention without the use of antibiotics and ecological competition in order to promote long-term genetic stability. These kill switches effectively caused strain evacuation not only in the murine bowel, but also during excretion, proving a strong, multi-layered live biotherapeutic architecture. In this way, such multilayered containments are such that they provide dynamic fail-safe control of the engineered probiotic with designed principles that reinforce clinical safety and regulatory compliance in the use of the same in treating human beings. Multi-input logic gates are also being used to control the activation of the engineered probiotics under strictly specified wound site conditions, like hypoxia, ROS accumulation, or high concentrations of inflammatory cytokines, to limit off-target activation of the engineered probiotics in healthy tissues [34, 225]. The result of this modular response design is that probiotics can only express their therapeutic payloads in response to pathological conditions therefore limiting operational scope and limiting exposure to the rest of the body. Moreover, genetic insulation measures (i.e. the application of synthetic plasmids with minimized homologies to natural microbial sequences, or insertion of therapeutic genes into the chromosome of RecA-deficient strains) can decrease the risk of horizontal gene transfer to native microbiota [345, 346]. This is so since RecA has a direct involvement in both the homologous recombination and SOS response by which the incorporation and exchange of foreign DNA material is enabled; consequently, deletion of RecA can provide a minimalistic case of unwarranted genetic rearrangement and the recurring horizontally transmitted genetic exchange. Physical containment measures like hydrogel-based smart dressings or encapsulated formulations can also enhance site specificity and prevent bacterial dissemination. Fine-tuning such context-dependent responses demands advances in synthetic biology logic gates and multi-input sensing modules. The translation of therapeutic solutions into clinical practice requires established animal models of human wound healing, which can precisely reflect chronic and diabetic wound healing traits [31, 347]. Research using current preclinical models concentrates on healing in acute wounds but fails to replicate the multi-factorial elements of impaired healing conditions. The clinical approval pathway for engineered probiotics exists in its early stages while needing standardized testing approaches for biosafety verification, plus classification frameworks that determine engineered probiotics’ status as a drug, device, biologic, or combination products. International clinical adoption of engineered probiotics will require essential regulatory harmonization to become possible [348, 349]. Long-term stability remains a challenge because it affects performance consistency through the healing period and patient-to-patient interactions with their microbiome have their limitations along with challenges in smart dressing system integration. The next rational progression would be to join engineered probiotic modules with biosensing dressings that incorporate feedback-controlled release mechanisms and environmental perceptiveness [225, 350]. The convergence creates new opportunities for advanced wound treatment which provides individualized therapy through real-time dynamic adjustments using measurements of infection levels and tissue inflammation and regeneration markers. The solution demands combined efforts between synthetic biologists with materials scientists along with clinicians and regulatory experts and systems engineers. Investments into implementing smart engineered probiotic platforms in trauma care treatments are justified by their significant therapeutic value although clinical deployment requires extensive development work.

**Figure 8:**
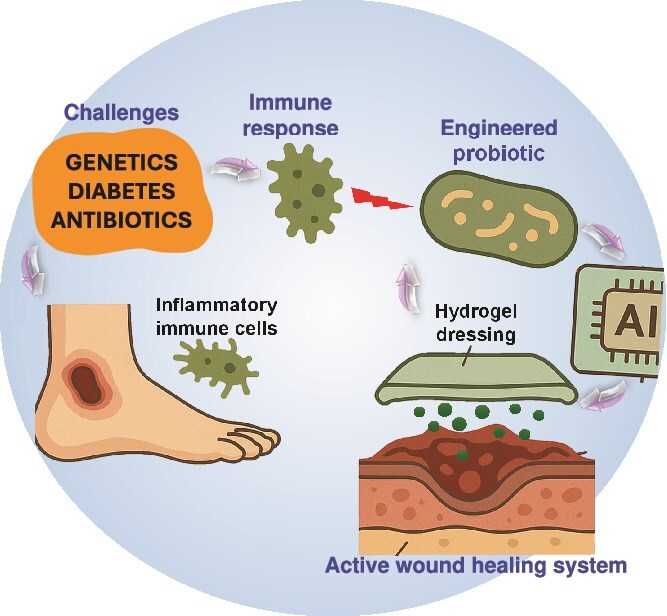
Conceptual framework of an intelligent engineered probiotic system for wound healing. This figure summarizes the difficulties of utilizing engineered probiotics in the management of chronic wounds, including issues of host heterogeneity, inflammation and exposure to antibiotics, and one future generation wound healing approach incorporating engineered probiotics into smart dressings embedded in hydrogels. Such dressings can detect local signals (e.g. cytokines, pH, ROS) and respond in an adaptive therapeutic manner (e.g. suppress inflammation, induce angiogenesis) in addition to being able to interact with host immunity. These systems, working with an AI-based biosensing platform are potentially able to dynamically adjust therapeutic output, which marks a paradigm change toward dynamic, responsive, and personalized treatment of trauma

Future smart wound healing systems might have wearable biosensors embedded into wound dressings themselves, so that their concept can actually be converted to the real world. A general picture of this conceptual framework is given in Figure 8. The figure not only depicts the integration of biosensing, engineered probiotics, and the AI-assistance of delivery systems, but it also provides a systems-level view of the interaction between these components in order to address issues of immune and microbial challenges in chronic wounds. The figure reflects the interaction between some major factors that influence poor healing processes, i.e. inflammation, antibiotic resistance, and diabetes, and proposes a smart hydrogel-based dressing as a core intervention system. At its heart, it involves the use of engineered probiotics that are embedded within the hydrogel, and can sense biomarkers in wounds, and provide targeted treatments (e.g. VEGF, IL-10). Surrounding this central unit are the real-time biosensors, which monitor pH, ROS, and the level of cytokines—the early indicators of the healing status or deterioration. These data are wirelessly transferred to a system connected to the cloud, where the AI algorithms make sense of the trends and make therapeutic decisions, such as activating inducers—to turn on gene circuits in the probiotics. By visualizing this integration in a systems-oriented way, Figure 8 raises the concept of this integration from individual innovation of components toward a holistic and closed-loop therapeutic ecosystem. This synthesis is a major departure from traditional passive wound dressings, which propose instead a programmable, feedback-controlled intervention model able to adapt to real-time physiological cues. The implications for the field are significant, especially for chronic wound management, where delayed healing, fluctuating immune responses, and biofilm persistence often call for dynamic and personalized solutions. These miniature sensors would constantly monitor such fundamental biological indicators as temperature (which may indicate inflammation or infection), changes in pH (wound deterioration), ROS (associated with chronic inflammation), and cytokine levels (needed to see how immune cells are responding to a wound site) [315, 351–356]. Collectively, the markers provide a real time update of the wound healing or lack of it. For instance, Kalasin and colleagues came up with a FLEX-AI bandage system that combines a pH-sensitive hydrogel dressing with a radio-frequency sensor and a deep neural network (ANN) that can tell what healing stage the wound is in—fast, slow, or not healing at all—with ~95% accuracy [357]. This intelligent dressing is not merely to do with measuring data; it transmits that information wirelessly and assists with hosting treatment options through the use of artificial intelligence (AI). The other one comes from the team of Mimee, who has developed a device that can be swallowed (IMBED), combining engineered probiotics with ultra-low-power wireless electronics [358]. Such designed probiotics light up when exposed to blood in the GI, and that message gets transmitted to a phone or computer. It was able to detect GI bleeding in pig tests with 100% accuracy in <2 hours, a good indication of the successful collaboration of biology and electronics inside the body. Once these biosensors collect data, they have the capability of sending the data wirelessly (such as through Bluetooth) to a phone or cloud system, where AI comes into play to then interpret trends (such as a sudden decrease in PH or increase in ROS) and determine what phase of healing the wound is stuck in. However, AI does not only observe, but it can also be coded to act. As soon as they realize they need help, the system may switch engineered probiotics to deliver therapeutic molecules such as VEGF (promotes blood vessel formation) or IL-10 (decrease inflammation). The designed probiotics are constructed using synthetic gene circuits which turn themselves on when they detect the presence of certain chemical inducing agents; serving as the chemical on buttons or switches. In view of the study of Liu *et al.* to show that neural probes containing integrated microstructures and microfluidic channels designed to locally deliver the anti-inflammatory drug minocycline to suppress substantial glial and microglial inflammatory responses, to identify a programmable, drug-delivery-based strategy to improve upon conventional long-term biocompatibility—an approach that may be adapted for AI-driven, on-demand therapeutic modulation in wound healing [359]. Therefore, the sensors as well as these inducers could be enclosed within microfluidic chambers in the bandage, which can hold the devices. When the AI comes to the conclusion that it is time to use it, it opens a microvalve and pours a bit of inducer. Once that molecule enters the hydrogel, it turns on the engineered probiotics that deliver the correct treatment at the appropriate moment. Now, some engineered probiotics can respond on their own to things like ROS or inflammatory cytokines—but they cannot do timed or programmable control. That is where AI-controlled systems shine. By relating sensing, data transmission, and AI-based decisions to the real behavior of probiotics, the smart healing platform is designed that be accurate, responsive, and more capable than passive wound care can be provided. While the conceptual integration between biosensors, AI and the creation of new probiotics is an exciting vision, technical feasibility is in consideration. Wearable biosensors that can accurately measure certain parameters (e.g. pH, ROS, cytokines) are already in active development, and a few prototypes (e.g. FLEX-AI) have demonstrated promising preclinical performance [357]. However, long-term stability, compatibility of the embedded electronics with living tissues and interference from the exudates of the wound remain technical issues. Similarly, while IMBED shows that engineered probiotics can interface with electronics within the body, as such systems are translated to non-sterile, chronic wound environments, additional challenges associated with microbial competition, nutrient availability and colonization efficiency are presented [358]. On the AI front, machine learning models for wound biomarker interpretable AI are receiving rapid development, but must include extensive datasets to improve patient population generalizability. Current smart bandage systems are still in the trial stages of experimentation or in early clinical trials. Realistically, integrated AI–biosensor–probiotic wound systems may require 5–10 years before reaching routine clinical deployment, depending on regulatory pathways, manufacturing scalability, and clinical validation [267, 268]. Therefore as these smart healing platforms have groundbreaking potential, their development should proceed in parallel with good engineering validation and clinical prototyping and regulatory alignment to assure translational success.

Regulatory-wise, the current FDA and EMA regulation seems complicated to classify and develop engineered live biotherapeutics. FDA clarifies controversial terms as follows, or rather conceptualize LBPs as biological products that include living organisms (not vaccines) with the purpose of disease prevention or treatment. They fall under (21 CFR Part 312) IND requirements that require meeting chemistry, manufacturing, and control (CMC) standards that the FDA took a closer look at in its recently published 2016 guidance [267]. In the case of recombinant LBPs, especially, there will be a requirement and need to have lengthy documentation on the nature of the strain, genetic manipulations, biological functioning, and possible risks in the environment, such as horizontal gene transfer, virulence factors, and the capability of microbial persistence. On the same note, the EMA guideline on recommendations regarding genetically modified different cells (EMA/CAT/GTWP/671639/2008) defines severe requirements in terms of the advanced therapy medicinal products (ATMP) guidance [268]. These involve characterizations of the gene vectors in detail, evaluation of transduction efficiency, risk of insertional mutagenesis and environmental risk assessment. Despite this rule formally excluding bacteria, the same principles do cover engineered probiotics constructed on eukaryotic chassis or with elaborate genetic circuits. Importantly, the EMA promotes an approach that is risk-based and allows adoption of development protocols according to the complexity of the product, the source material, and therapeutic indication. The major barriers to commercialization are implementation of stringent biosafety measures, namely kill switches, auxotrophic circuits to prevent any off-targets and environmental escape [267, 360]. Such mechanisms have to reveal genetic stability as well as effective performance over product shelf life and clinical use conditions. Other problems are the profiling of host-microbiome interactions, reduction of immunogenicity, and the development of strain persistence and clearance profiles. Notably, advanced and ongoing communication with regulatory organizations (FDA pre IND conferences; EMA scientific advice processes) is of great importance. This promotes consensus regarding product typology (biologic vs. drug vs. combination product), acceptable manufacturing tolerance and validation of clinical models (e.g. chronic wound or inflammatory disease status). In addition to that, extended stability studies, good manufacturing practices, and harmonized safety/efficacy standards have a vital role to play in translating preclinical research to practical implementation. Universal microbiome interaction testing and surrogate measures of potency will also help approve drugs faster, as they will lead to comparable standards of therapeutic action.

## Conclusions

The scientific perception that bacteria exist solely as wound pathogens is experiencing rapid transformation. Scientific evidence now demonstrates that specific bacterial strains which include natural commensal bacteria as well as probiotics and engineered probiotics, function as active partners during trauma recovery processes. The bacteria engage with host defense mechanisms while controlling inflammation and aiding the body’s healing processes, as well as helping to protect from resistant microorganisms. The paradigm change disrupts long-run antimicrobial approaches while creating opportunities for microbiome-based regenerative techniques. The review creates an extensive body of knowledge that joins fundamental understanding of immunological processes to present-day advancements in synthetic biology and materials science. By tracing the journey from natural immune modulation by skin-resident microbiome to the cutting-edge applications of engineered probiotics, we highlight how probiotic allies can be rationally designed to enhance wound healing. The study presents fundamental descriptions of microbial immunoregulation with systematic exploration of probiotic intervention types and innovative biochemical engineering techniques that combine material-based wound care with immune system enhancement through techniques from genetic modification to surface modification to membrane vesicle applications. Engineering microbial therapeutics for clinical use requires strong collaboration between researchers in microbiology along with immunologists and bioengineers and synthetic biologists along with medical practitioners. Ongoing future research focusing on complex and chronic wounds needs to demonstrate the medical viability of microbial therapeutic approaches. Multi-field creativity enables bacterial development into dependable therapeutic partners to shape intelligent bioresponsive treatments that will be used in future trauma and healing medicine.
